# γδ T cells: bridging innate and adaptive immunity in development, tissue residency, and disease

**DOI:** 10.3389/fimmu.2026.1920627

**Published:** 2026-10-06

**Authors:** Jiacheng Lou, Ning Zou

**Affiliations:** Department of Pediatrics, the Second Hospital of Dalian Medical University, Dalian, China

**Keywords:** immunotherapy, innate-like lymphocytes, tissue-resident T cells, γδ T cells, γδ TCR complex

## Abstract

γδ T cells are a unique lymphocyte lineage that bridges innate and adaptive immunity through MHC-unrestricted recognition of phosphoantigens, lipids, and stress-induced proteins. Their tissue-resident subsets, shaped by Vγ/Vδ chain usage, play essential roles in barrier surveillance, tissue homeostasis, and host defense.This review synthesizes recent advances that fundamentally reshape our understanding of γδ T cell biology. We highlight two conceptual frameworks: (1) dual recognition modalities of γδ TCRs, distinguishing structurally resolved ligand interactions from functionally supported recognition; and (2) a developmental wave model in which TCR signal strength and transcriptional networks program effector fates. Mouse and human studies are presented in dedicated species-specific sections and tables, with explicit attention to species-specific nomenclature and evidence levels.We cover γδ T cell development, tissue-resident programs, antigen recognition mechanisms (including butyrophilin-mediated phosphoantigen sensing, antibody-like foreign antigen recognition, and intracellular self-protein recognition), and the dual roles of γδ T cells in infection, cancer, and chronic inflammation. Therapeutic applications—adoptive transfer, CAR-engineered cells, and extracellular-vesicle vaccines—are critically appraised against current clinical evidence.Finally, we identify major unanswered questions: the complete γδ TCR ligand repertoire, the mechanisms maintaining tissue residency, and the translational gaps between mouse models and human disease. This review provides a structured framework for understanding γδ T cells as pivotal connectors of innate and adaptive immunity.

## Introduction

γδ T cells are a distinct lymphocyte lineage at the interface of innate and adaptive immunity. Unlike αβ T cells, their TCR—composed of γ and δ chains—recognizes lipids, phosphoantigens, and stress-induced proteins in an MHC-unrestricted manner (1–3), enabling rapid responses to stress, infection, and transformation (3). Their evolutionary conservation underscores their fundamental role in host defense (4).

A defining feature is tissue tropism: subsets defined by Vγ/Vδ usage localize to specific sites. Throughout this review, we use the Heilig–Wilson nomenclature for mouse Vγ chains (Vγ1–Vγ7) and the IMGT-derived nomenclature for human Vγ chains (Vγ2–Vγ11, corresponding to TRGV2–TRGV11); equivalent numbering does NOT imply homology between species (e.g., mouse Vγ5 and human Vγ5 are distinct genes). In mice [Heilig–Wilson], Vγ5Vδ1 DETCs reside in skin and Vγ7+ IELs in intestinal epithelium (5, 6). In humans [IMGT], Vδ2+Vγ9+ cells dominate blood (also denoted Vγ9Vδ2), while Vδ1+ cells are enriched in epithelia (8–10), reflecting specialized roles in tissue homeostasis and barrier integrity (7).

γδ T cells undergo intrathymic effector programming, generating pre-committed IFN-γ- or IL-17-producing subsets influenced by TCR signal strength and transcription factors (12–16). IL-17-producing γδ T cells (γδT17) develop primarily in fetal/perinatal life, forming long-lived, self-renewing tissue populations (8, 9). In humans, fetal thymic waves produce innate-like γδ T cells persisting into adulthood (10, 11).

Functionally, γδ T cells mediate protective immunity against diverse pathogens via cytotoxicity and cytokine production (12–15). In CMV and influenza, they exhibit adaptive-like memory (16–18). They contribute to tumor immunosurveillance (19–21), though certain subsets promote tumor progression via IL-17 (22–24). They also regulate tissue repair, wound healing, bone regeneration, and adipose maintenance (25–28), while dysregulated responses drive psoriasis, multiple sclerosis, and IBD (29–34).

Beyond their autonomous effector functions, γδ T cells serve as immunological bridges between innate and adaptive immunity. They act as professional antigen-presenting cells—human Vγ9Vδ2 and Vδ1+ subsets upregulate HLA-DR, CD80/86, and MHC class II following activation, processing and presenting soluble antigens to αβ T cells and priming adaptive CD4+ and CD8+ T-cell responses (see **Table 1**). γδ T cells also shape humoral immunity by promoting B-cell differentiation, germinal center reactions, and immunoglobulin class switching through cytokines (IL-17, IL-21, IFN-γ) and CD40L expression. Concurrently, they integrate upstream innate signals—IL-1β, IL-18, IL-23, and NKG2D ligands—from tissue-resident macrophages, dendritic cells, and stressed epithelia, translating these into coordinated effector outputs that recruit and instruct αβ T cells, NK cells, and myeloid populations. This bidirectional positioning—receiving innate instruction while licensing adaptive responses—makes γδ T cells a true cellular bridge, and the sections below trace how this bridging capacity is developmentally wired, anatomically deployed, and clinically exploitable. This unifying bridging framework is illustrated in **Figure 1**. Therapeutically, their MHC-independent tumor recognition and cytotoxicity make them attractive for cancer immunotherapy (35–37). Elucidating butyrophilin-mediated phosphoantigen presentation has opened new intervention avenues (38–41).

**Table 1:** γδ T cells in cancer and autoimmunity: metabolic and mechanistic drivers (mouse models, human correlative, and disease contexts).

Tissue	Cells	Functions	Citations
Cancer	Vγ7+ and Vγ1+ γδ T cells (CRC) (mouse)	Cytotoxic, IFN-γ-producing anti-tumor profiles in epithelium	(21)
Cancer	Vγ6+Vδ1+ γδ T cells (CRC) (mouse)	PD-1+ IL-17-producing pro-tumorigenic phenotype in tumors	(21)
Cancer	Glut1 inactivation (early)	Impairs glycolysis-dependent epithelium-resident γδ T cells, heightens tumor development	(21)
Cancer	Rorγt targeting (late)	Reduces tumor growth	(21)
Cancer	Decitabine (DNMTi)	Upregulates NKG2D ligands and death receptors on tumor cells; DNMTi-associated cytoskeleton signature stratifies lung cancer prognosis	(186)
Cancer	γδIFN vs γδT17 metabolic programming	γδIFN depend on glycolysis (Myc-driven); γδT17 engage OXPHOS; low glucose favors γδT17, high glucose enhances γδIFN antitumor function	(75)
Cancer	Pro-tumoral γδT17 in TME	High lipid uptake, expanded in obesity; glucose supplementation enhances γδIFN antitumor functions	(75)
Cancer	Palmitic acid (PA)/Oleic acid (OA)	PA suppresses Vγ9Vδ2 antitumor activity via CD36-mediated fatty acid regulation; OA restores function; PA/OA ratio correlates with HCC survival	(187)
Cancer	STING pathway	Tumor-derived cGAMP activates STING in γδ T cells (TBK1–Eomes–IFN-γ axis); Sting1−/− Vγ4 cells show reduced killing and increased metastasis; TGF-β downregulates STING	(188)
Cancer	BTN3A1/BTNL2	BTN3A1 overexpressed in tumors suppresses αβ T cells; anti-BTN3A1 + adoptive transfer shows maximal efficacy in ovarian cancer. BTNL2 blockade reduces γδT17-driven tumor growth	(24, 90)
Autoimmunity	Dermal γδ T cells	Constitutively express IL-23R and RORγt; dominant source of IL-17 driving epidermal hyperplasia in psoriasis	(29)
Autoimmunity	γδT17 metabolic reprogramming (psoriasis)	Switch to aerobic glycolysis; ACLY-ACC1-FASN axis supports *de novo* fatty acid synthesis; ACC1 inhibition ameliorates psoriatic inflammation	(122)
Autoimmunity	Tissue-derived metabolic cues	Epithelial lactate augments type-17 responses; GPR183 (oxysterol) required for dermal γδT17 maturation; CD69 controls AhR-IL-22 via LAT1	(76, 77, 206)
Autoimmunity	Neonatal antibiotic treatment	Dysregulates microbiota, increasing IL-22-producing γδ T cells and exacerbating experimental psoriasis	(207)
Autoimmunity	Adiponectin	Suppresses IL-17 from dermal Vγ4+ γδ T cells; deficiency results in severe psoriasiform inflammation	(208)
Autoimmunity	Aged skin γδ T cells	IL-17-skewed; contribute to chronic inflammation, delayed by IL-17 blockade	(130)
Autoimmunity	Effector memory Vγ9Vδ2 T cells	Acquire antigen-presenting characteristics (HLA-DR, CD80/86); present soluble antigens to CD4+ T cells in RA	(209)
Autoimmunity	Spondyloarthritis (SpA) γδ T cells	RORγt+T-betlo PLZF− TCRγδ-hi subset expanded in inflamed joints; cigarette smoke enhances citrullinated antigen reactivity (HLA-DR4)	(210, 211)
Autoimmunity	IL-1Ra–IL-1R axis	Vγ6+ CCR2+ γδ T cells are primary IL-17 producers in IL-1Ra-deficient joints; IL-1Ra normally suppresses IL-1R on γδ T cells	(33)

**Figure 1:**
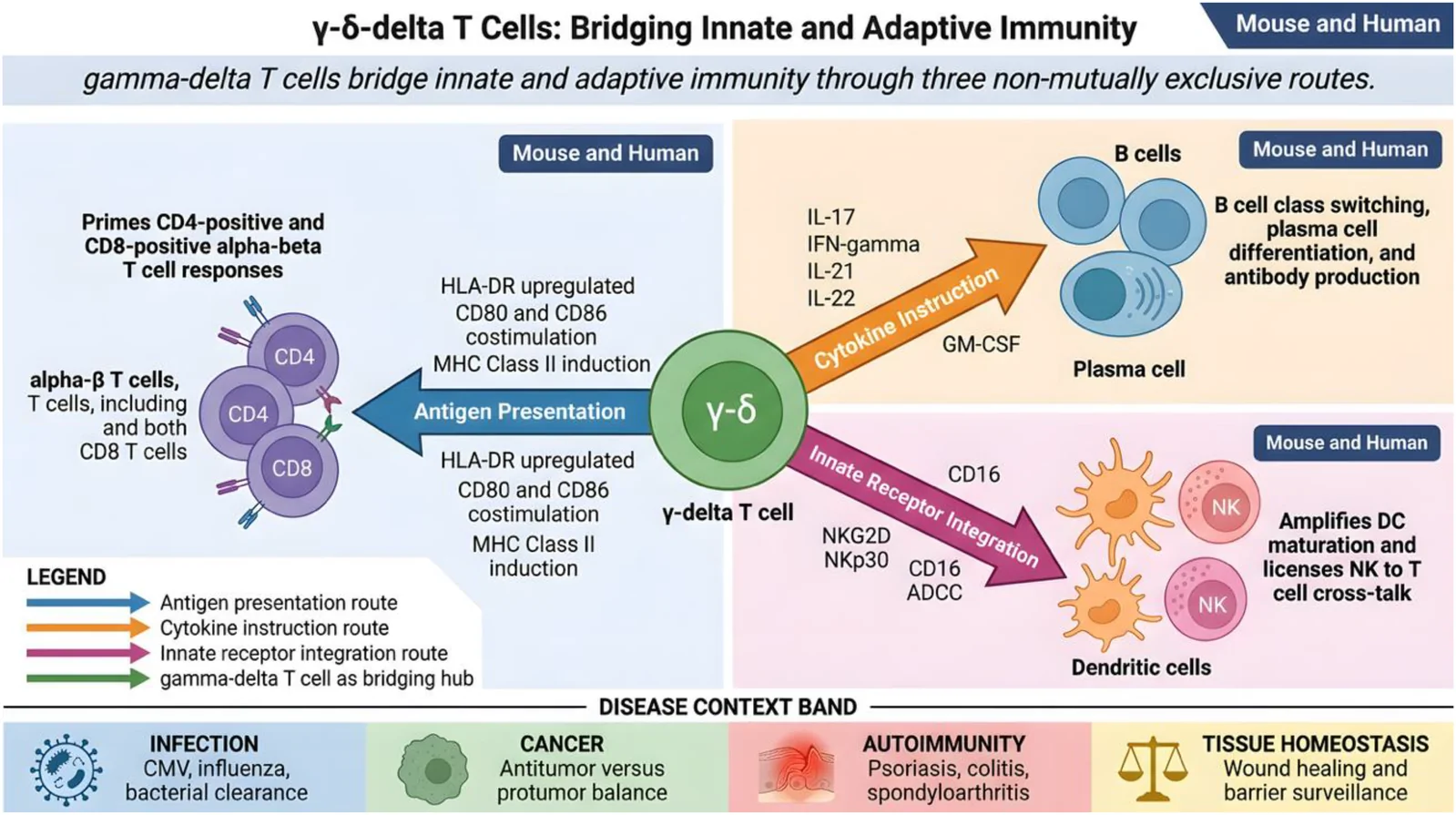
γδ T Cells: bridging innate and adaptive immunity. This new unifying figure visualizes the central conceptual framework of the review. A central green γδ T cell icon serves as the bridging hub, with three color-coded routes radiating outward to distinct immune compartments. Route 1 (blue, left) -- Antigen Presentation: γδ T cells act as professional antigen-presenting cells (HLA-DR upregulated, CD80/CD86 costimulation, MHC Class II induction) and prime CD4+ and CD8+ αβ T cell responses. Route 2 (orange, top-right) -- Cytokine Instruction: γδ-derived IL-17, IFN-γ, IL-21, IL-22, and GM-CSF instruct B-cell class switching, plasma-cell differentiation, neutrophil recruitment, and ILC3 modulation. Route 3 (magenta, bottom-right) -- Innate-Receptor Integration: γδ T cells integrate innate-receptor inputs (NKG2D, NKp30, CD16-mediated ADCC) to amplify dendritic-cell maturation and license NK-to-T-cell cross-talk. A bottom disease-context strip situates the bridging framework across four contexts -- Infection (CMV, influenza, bacterial clearance), Cancer (antitumor versus pro-tumor balance), Autoimmunity (psoriasis, colitis, spondyloarthritis), and Tissue Homeostasis (wound healing and barrier surveillance). The top banner emphasizes that the three bridging routes are non-mutually exclusive. Together, this figure serves as the conceptual summary of the entire review.

## Development and subset diversification of γδ T cells

### Developmental waves and ontogeny in mice

The murine ontogeny of γδ T cells features layered immune system development with distinct fetal and adult progenitor waves. The first thymic-settling progenitors generate Vγ3+ DETCs, while a second multipotent wave maintains lifelong thymopoiesis without contributing to embryonic γδ T cell pools (42) (see **Table 2**). In the cited murine study (8), fetal-derived γδT17 cells — notably Vγ6+ subsets — are generated primarily during a functional embryonic wave and persist as long-lived, self-renewing cells in adult tissues; this ontogenetic pattern may be subset-specific rather than universal. An innate Vγ4+Vδ5+ population with germline-encoded TCR rearrangements is restricted to CCR6+CD27− cells generated only in the fetal thymus (43). The RNA-binding protein Lin28b, specifically expressed in fetal HSCs, reprograms adult progenitors for innate-like lymphocyte development by post-transcriptionally blocking let-7 miRNA biogenesis, de-repressing Hmga2 and Igf2 (44). A perinatal wave of innate IFN-γ-producing CD8αβ+ γδ T cells populates neonatal tissues and persists into adulthood (46).

**Table 2:** Key features of γδ T cell development and subset diversification (murine, human, and other-species sections).

Features	Functions	Citations
Mouse (murine studies)		
First thymic-settling progenitors	T-lineage restricted; generate Vγ3^+^ DETCs	(42)
Second multipotent wave	Maintains lifelong thymopoiesis; no contribution to embryonic γδ T cell pools	(42)
γδT17 cells	IL-17-producing; generated primarily (not exclusively) during a functional embryonic wave in the cited murine study; persist as long-lived, self-renewing cells in adult tissues	(8)
Innate Vγ4^+^Vδ5^+^ population	Germline-encoded TCR rearrangements lacking N-nucleotides; restricted to CCR6^+^CD27^-^ compartment; generated only in fetal thymus	(43)
Lin28b	RNA-binding protein; specifically expressed in fetal HSCs; reprograms adult progenitors for innate-like lymphocyte development; blocks let-7 miRNA biogenesis; de-represses Hmga2 and Igf2	(44)
Ectopic Lin28 expression in adult HSPCs	Skews lymphocyte repertoire; >15-fold increase in γδ-TCR^+^CD3^+^ thymocytes; primarily CD4 SP Vγ1.1^+^Vδ6.3^+^ innate-like cells expressing PLZF	(44)
Perinatal innate IFN-γ-producing CD8αβ^+^ γδ T cells	Populate neonatal tissues (~24% spleen, ~13% lungs, ~35% small intestine, ~52% lymph nodes); persist into adulthood; respond to tumor/infection by expanding and producing IFN-γ	(46)
Immature γδ thymocyte subtypes	Three molecularly distinct subtypes: Vγ3^+^ (DETC precursors), Vγ4^+^/Vγ2^+^ (IL-17 programmed), Vγ1^+^/Vγ6^+^/Vγ5^+^ subsets; each with unique transcription factor modules	(50)
Immature Vγ2^+^ subset	Global gene expression profile radically different from other immature γδ subsets	(50)
Human		
STAG2/LMO2 subtype in human γδ T-ALL	Up-regulation of fetal genes; suggests fetal HSC origin; relevance of LIN28/let-7 pathway	(45)
Twin cohort heritability of innate-like T cells	γδ T cells show far less heritability than conventional CD4^+^/CD8^+^ T cells; high variation from shared environmental influences	(48)
Vδ2^+^Vγ9^+^ vs Vδ1^+^ heritability	Vδ2^+^Vγ9^+^ cells show higher heritability; Vδ1^+^ cells display strong shared environmental influence, likely imprinted during childhood	(48)
Human tissue γδ T cell frequencies across ages	Children harbor greater proportions in blood, lung, jejunum, spleen; frequencies decrease with age	(49)
Early-life Vδ1 subsets	Clonally diverse; predominate with naïve and tissue-repair functions	(49)
Cytolytic Vδ2 subsets	Populate blood, spleen, lungs; with age undergo clonal expansion and elevated cytolytic signatures; predominate in adult blood	(49)
Other species (evolutionary perspectives)		
Naked mole-rat public invariant Vγ4-2/Vδ1–4 TCR	Generated by short-homology-repeat-driven rearrangements; expressed by NK-like cytotoxic γδ T cells throughout adult life in thoracic and cervical thymi	(47)
Lamprey VLRC^+^ and VLRA^+^ lymphocytes	Express orthologues of gnathostome γδ and αβ T cell differentiation genes; indicate two primordial T-cell lineages existed ~500 million years ago	(4)

Vγ5+ DETC progenitors induce Aire+ mTECs in a Rank-Rankl-dependent manner (51). Rank-deficient mice exhibit dramatic reduction in mature DETCs, linked to Skint1 expression (51). Aire-deficient mice show expanded perinatal γδT17 cells; Aire controls Il7 expression in mTECs to regulate this compartment (52). Non-canonical ERK action specifies αβ versus γδ fate (53). HEB controls fetal γδT17 specification (54). A core innate effector program is shared across NK cells, iNKT cells, and γδ T cells (55). SOX4 and SOX13 directly regulate Rorc and Blk (56). CRISPR/Cas9 dissection of Zbtb16 reveals a critical +21/23 sub-region with Runx1 binding sites essential for innate lymphoid lineage development (57). mTORC1 and MYC constitute a reciprocal feed-forward loop; RAPTOR deficiency causes ~10-fold reduction of DP cells and reciprocally promotes γδ T cell development (58).

### Developmental waves and ontogeny in humans

In humans, γδ T cell development is organized in gestational-age-dependent waves, and the murine layered-ontogeny framework can be only partially extrapolated. The LIN28/let-7 pathway is relevant to human γδ T-ALL, where a STAG2/LMO2 subtype exhibits up-regulation of fetal genes (45).

Early-life thymectomy leads to persistence of fetal-derived effector γδ T cells with reduced repertoire diversity (81). γδ T-ALL accounts for ~9.6% of T-ALL with inferior survival (45).

Twin cohort studies reveal that innate-like T cell populations show far less heritability than conventional T cells, with high variation ascribed to shared environmental influences (48). Human tissue analysis from 176 donors confirms that children harbor greater γδ T cell proportions in blood, lung, jejunum, and spleen, with frequencies decreasing with age (49). Early-life Vδ1 subsets predominate with naïve and tissue-repair functions, while cytolytic Vδ2 subsets populate blood and spleen; with age, both undergo clonal expansion and elevated cytolytic signatures (49).

### Evolutionary conservation of developmental programs

The naked mole-rat illustrates evolutionary conservation with a public invariant Vγ4-2/Vδ1–4 TCR expressed by NK-like cytotoxic γδ T cells throughout adult life (47). In lampreys, VLRC+ and VLRA+ lymphocytes express orthologues of gnathostome γδ and αβ T cell differentiation genes (4). The layered ontogeny, effector programming, transcriptional regulation, and human-mouse divergence covered in this section are summarized in **Figure 2**.

**Figure 2:**
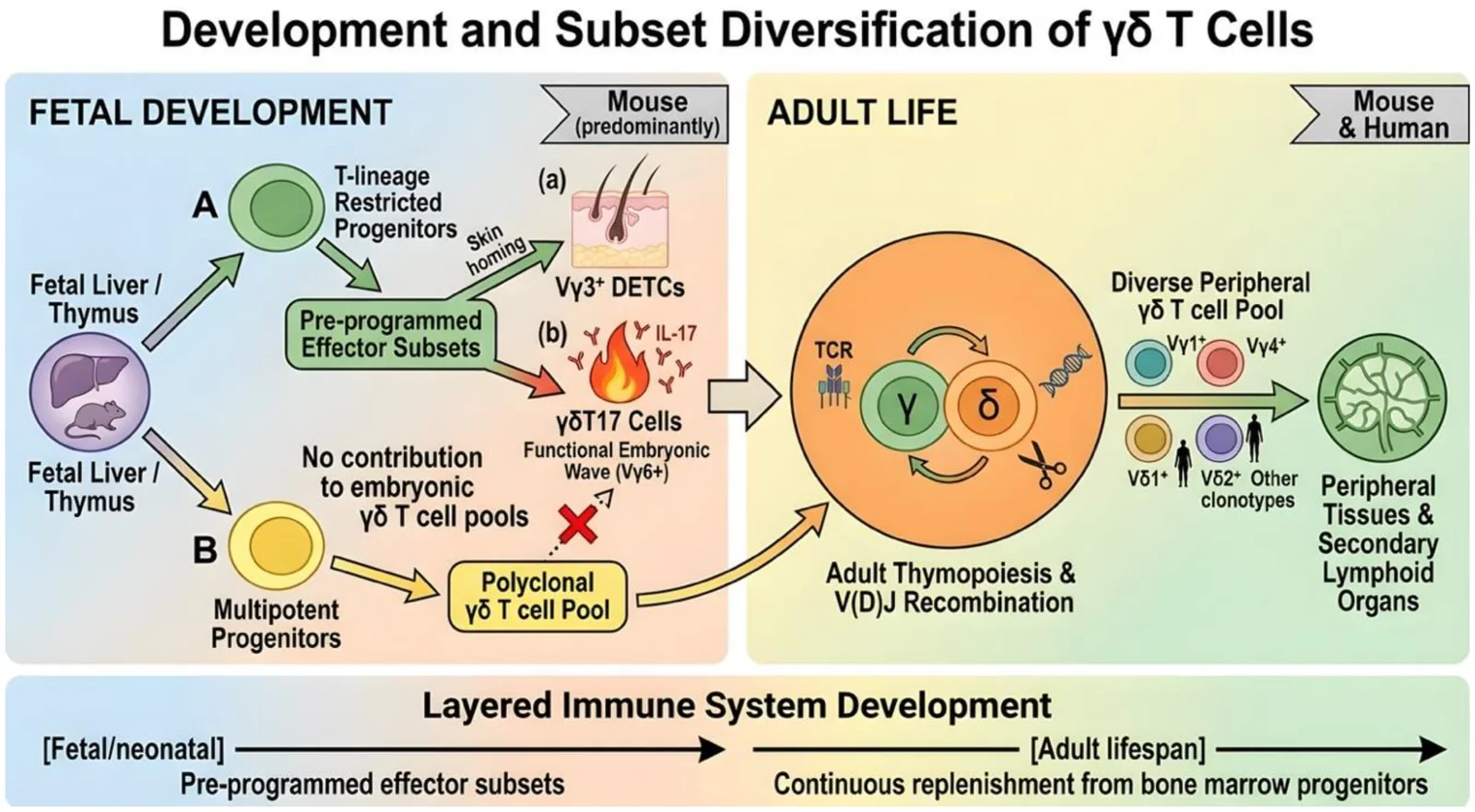
Development and subset diversification of γδ T cells schematic summarizing the layered ontogeny of γδ T cells, spanning fetal waves of pre-programmed effector subsets (Vγ5+ dendritic epidermal T cells, Vγ6+ γδT17, Vγ1+ and Vγ7+ subsets in mice; first-trimester Vδ2+ and second-trimester Vδ1+ waves in humans) and the transition to a polyclonal, TCR-diverse γδ T cell pool generated through lifelong adult thymopoiesis. The figure is organized into four sections corresponding to the restructured Development subsection: (i) Developmental Waves, (ii) Effector Programming (IFN-γ, IL-17, cytotoxic programs), (iii) Transcriptional Regulation (SOX13, RORγt, TCF1, BCL6, IRF4, STAT networks), and (iv) Human -Mouse Divergence. Distinct color codes distinguish T-lineage -restricted (green), multipotent (yellow), and perinatal γδT17 (red) developmental intermediates. Species banners identify mouse-predominant versus combined mouse/human contexts. Mouse Vγ chains are named using the Heilig -Wilson convention; human Vγ/Vδ chains follow IMGT.

### Effector programming: TCR signal strength model in mice

The pre-wired γδT17 program and TCR-dependent effector programming are not contradictory but reflect a quantitative signal-strength model. In the absence of strong TCR engagement, DN1d SOX13+ progenitors execute a default γδT17 transcriptional program (9). When TCR signaling exceeds a threshold—set by TCR–ligand affinity, co-receptor engagement, and developmental window—alternative programs are activated: strong signaling skews toward γδT1/IFN-γ commitment via Id3/Egr2 induction (50, 59), whereas low TCR signaling plus IL-4 elicits CD8αβ+ innate IFN-γ-producing γδ T cells (46). CD3DH mice with titrated TCR signal strength directly demonstrate this dose–response relationship (59). Thus, pre-wiring represents the TCR-independent default pathway that is modulated, not abolished, by TCR signals—reconciling the two models.

Within the thymus, transcriptomic analyses segregate immature γδ thymocytes into three molecularly distinct subtypes: Vγ3+ (DETC precursors), Vγ4+/Vγ2+ (IL-17 programmed), and Vγ1+/Vγ6+/Vγ5+ subsets (50). CD3DH mice demonstrate that TCR signal strength within specific developmental windows determines proinflammatory subset generation (59). Noncanonical ERK action through its DEF-binding pocket is essential for γδ T cell lineage specification (53). mTORC1 couples microenvironmental cues with metabolic programs to orchestrate reciprocal αβ/γδ T cell development via ROS control (58). CD8αβ+ γδ T cell development requires low TCR signaling and IL-4-mediated Eomes upregulation (46). T cell-specific deletion of GFAT1 impairs αβ-T cell ontogeny while elevating γδ-T cell numbers (60).

TCR-negative SOX13+ progenitors within DN1d thymocytes precociously express RORγt, cMAF, BLK, and SCART2 prior to TCR expression, indicating pre-wired γδT17 programming (9). ~40% of DN1d cells express a near-complete γδT17 program, and γδT17 lineage programming proceeds independently of γδTCR signaling (9). The γδT1 lineage is promoted by elevated Id2, Id3, and Egr2 (50). Skint-1 engages Vγ5+Vδ1+ thymocytes to induce Egr3-mediated IFN-γ potential while suppressing IL-17 capacity (62). TOX stabilizes TCF-1 to maintain development balance; TOX deficiency causes enhanced γδT17 differentiation and fatal autoimmune hepatitis (63). Id3 is essential for normal development (64, 65). CD8αβ+ γδ T cells represent a committed innate IFN-γ-producing lineage (46).

γδ thymocyte maturation involves two-step effector programming: establishment at the immature stage and priming at the mature stage (50). Mature Vγ2+ cells super-induce Il23r (~100-fold) and Il1r1 (50). Seven discrete developmental stages branch into three pathways: adaptive-like naïve, IFN-γ-producing γδT1, and IFN-γ/IL-4-co-producing γδNKT cells (66). scRNA-seq demonstrates that γδ T, iNKT, and MAIT cells share analogous effector subsets maturing via shared, branched differentiation pathways (67). SOX13+ DN1d progenitors establish that effector lineage commitment can occur cell-intrinsically (9).

### Effector programming in humans

In humans, effector programming has been mapped primarily through gestational timing and TCR-chain usage rather than by experimentally titrated TCR signal strength. Human γδ TCR constant domains tune antigen sensitivity: Cγ1-containing TCRs achieve higher activation, with TRGC1-expressing cells skewing toward cytotoxic differentiation (61). Human fetal γδ thymocytes are committed to effector fates in wave-like patterns depending on gestational age (11). A three-stage developmental pathway exists for Vγ9Vδ2 T cells postnatally (80).

### Transcriptional and epigenetic regulation in mice

Transcriptional control of γδT17 commitment involves SOX4 and SOX13 directly regulating Rorc and Blk, while TCF-1 and LEF1 counter SOX proteins (56). A spontaneous Sox13 mutation causes selective Vγ4+ γδT17 deficiency and protection from psoriasis (68). c-Maf is a universal regulator essential for commitment and maintenance of all γδT17 cells; Maf deficiency causes complete loss of RORγt+ CCR6+ IL-17A+ γδ T cells and 6-fold higher fungal burden upon Candida infection (69). HEB is required for specification of fetal-derived CD73+ γδT17 cells (54). The Notch-Hes1 pathway is critically involved (70), as are NF-κB members RelA and RelB (71). BTLA limits IL-17 production and controls homeostasis (72).

Epigenetic programming reinforces effector fates: CD27+ cells are committed to Ifng with a closed Il17 locus, while CD27− cells display permissive chromatin at both loci (73). miR-146a acts as a thymically imprinted brake on IFN-γ in CD27−CCR6+ cells (74). Distinct metabolic programs are thymically established: IFN-γ+ cells depend on glycolysis, IL-17+ cells rely on OXPHOS and lipid metabolism (75). GPR183 senses oxysterols required for dermal γδT17 maturation (76).

### Transcriptional and epigenetic regulation in humans

Human data on the transcriptional and epigenetic control of γδ T cell effector fates remain sparse. CD69 controls AhR-dependent IL-22 via L-tryptophan uptake regulation (77). As noted above, the LIN28/let-7 pathway and fetal-gene programs are implicated in human γδ T-ALL, indicating that fetal-like transcriptional programs can be re-activated in human disease.

### Human-mouse divergence in development

Human evidence: Post-thymic human γδ T cell maturation across the lifespan, mapped via high-dimensional profiling of 176 donors, identifies six transcriptionally distinct clusters: Naïve, Tissue-adapted, Cytolytic/effector, and Repair modules (49). Adults show eight functional clusters dominated by effector and cytolytic profiles (49).

Murine evidence: γδ T cells promote Tfh cell differentiation; TCRδ−/− mice exhibit impaired germinal center formation and 50% reduction in Tfh cells (78). Peripheral γδT17 and γδIFN subsets show profound transcriptional divergence, with 49% of genes differentially expressed (79). CD6 and Themis are novel signature genes selectively enriched in γδIFN cells (79). CD8αβ+ γδ T cells account for almost half of IFN-γ-producing TILs in breast cancer (46).

## Ligand recognition and activation mechanisms

Three architecturally meaningful structural differences distinguish γδ TCR–CD3 complexes from αβ TCR–CD3 complexes, as resolved by recent cryo-EM studies (90–92). FIRST, the extracellular domains of γδ TCRs exhibit ~30° rotational freedom relative to CD3 subunits, whereas αβ TCR ECDs are constrained by extensive constant-domain–CD3 interfaces. SECOND, in γδ TCRs the constant domains lack these stabilizing contacts, so the ECDs are tethered to CD3 principally via their transmembrane helices. THIRD, cholesterol molecules occupy inter-chain hydrophobic clefts in the γδ TCR–CD3 complex (~70-fold enrichment over cholesterol sulfate) and act as allosteric inhibitors of signaling—a regulatory feature absent from αβ TCRs. Together these features endow γδ TCRs with mechanosensing capability that is structurally distinct from, rather than simply “more flexible” than, αβ TCRs. A 3.01 Å structure of an MR1-reactive human Vγ8Vδ3 TCR–CD3δγϵ2ζ2 complex demonstrated considerable conformational heterogeneity, with TCR-γδ extracellular domains tethered to CD3 subunits only by their transmembrane regions (87). This flexibility stems from lack of stabilizing contacts between γδ TCR constant domains and CD3 ECDs (88, 89). Cholesterol molecules wedge into hydrophobic clefts between chains; cholesterol is ~70-fold more abundant than cholesterol sulfate in the complex and acts as an inhibitory regulator of TCR signaling (89). The Vγ5Vδ1 TCR–CD3 complex forms a dimeric architecture essential for activation, revealing Vγ-dependent assembly mechanisms (89). These three distinguishing structural features are illustrated in the left panel of **Figure 3**.

**Figure 3:**
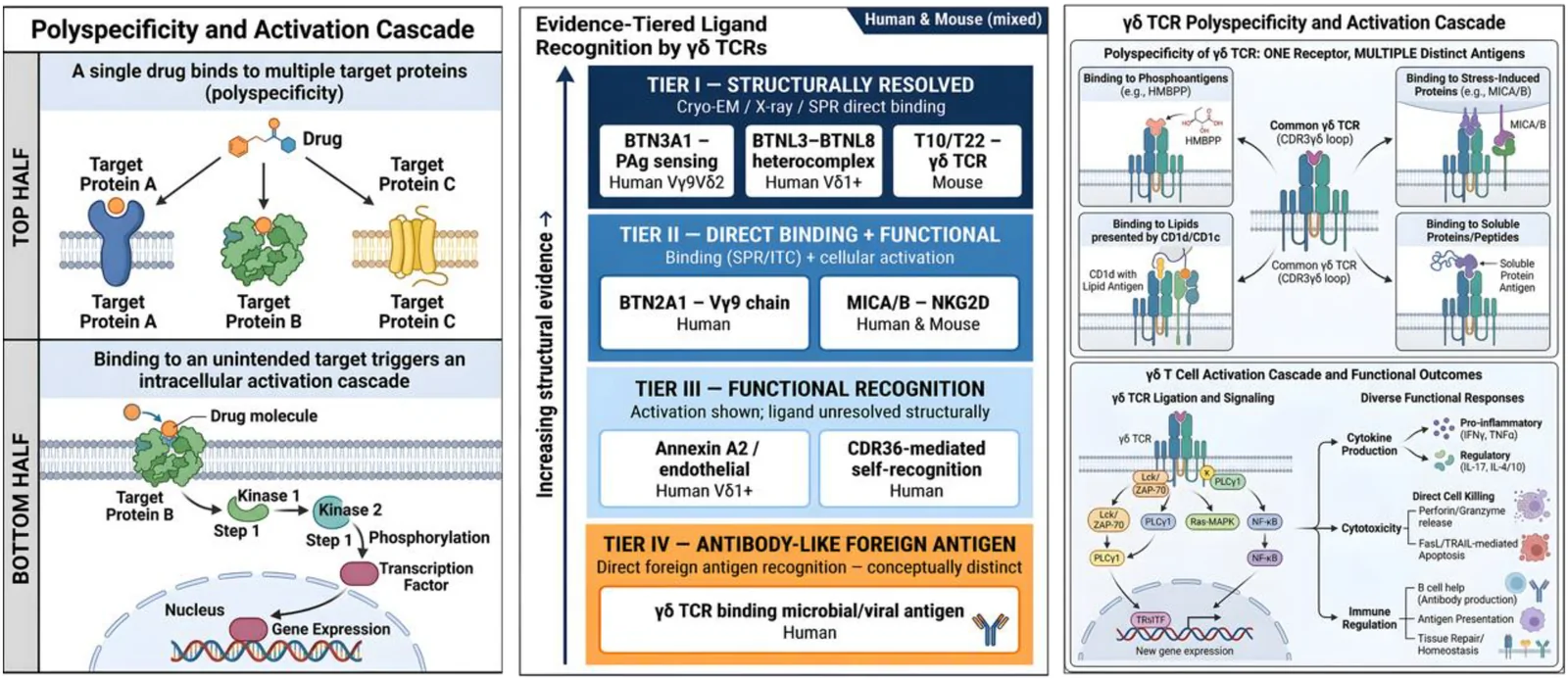
Ligand recognition and activation mechanisms of γδ TCRs composite figure integrating structural, functional, and conceptual advances in γδ TCR recognition, organized into three panels. Panel A (Structural Features) highlights the specific features that distinguish γδ TCRs from αβ TCRs, replacing the previous "rigid versus flexible" shorthand: (i) extracellular-domain rotational mobility (~30°), (ii) constant-domain -CD3 tethering restricted to transmembrane helices, and (iii) inter-chain cholesterol acting as an allosteric regulator. Panel B (Four-Tier Evidence Hierarchy) classifies γδ TCR -ligand interactions by evidence level: Tier I -- Structurally resolved (cryo-EM/X-ray/SPR), including BTN3A1 phosphoantigen sensing, the BTNL3/BTNL8 heterocomplex, and the classical murine T10/T22 system (97); Tier II -- Direct binding plus functional evidence, including BTN2A1 -Vγ9Vδ2 and MICA/B -NKG2D; Tier III -- Functional recognition only (ligand unresolved), including Annexin A2 and endothelial CDR3δ peptide recognition (111); Tier IV -- Antibody-like foreign antigen recognition (108), a conceptually distinct mode analogous to antibody -antigen engagement. Panel C (Polyspecificity and Activation Cascade) illustrates how a single γδ TCR can cross-recognize structurally distinct self-, microbial-, and viral-derived antigens (111) and links recognition to downstream activation outputs. Solid arrows denote direct receptor -ligand binding; dashed arrows denote functional/indirect relationships. Species banners identify the relevant context (human Vγ9Vδ2, human intestinal Vδ1+, or mouse) per panel.

### Butyrophilin-mediated recognition

To interpret BTN/BTNL-mediated γδ T cell recognition rigorously, we apply a three-tier evidence hierarchy throughout this section: Tier I — direct binding demonstrated by biophysical methods (SPR, ITC, or cryo-EM); Tier II — cellular activation via receptor engagement (e.g., reporter assays, cytotoxicity); Tier III — physiological relevance *in vivo* (genetic knockout, disease models). Findings supported by multiple tiers are reported as established mechanisms, whereas single-tier evidence is labeled as provisional. This tiered framework is applied throughout the section and visualized in the center panel of **Figure 3**.

BTN family members serve distinct roles in γδ T cell recognition that must not be conflated (see **Table 3**). BTN3A1 functions as an intracellular phosphoantigen sensor rather than a direct TCR ligand: its cytoplasmic B30.2 domain binds phosphoantigens (PAg), inducing an allosteric conformational change that promotes surface BTN3A1–BTN2A1 heterotetramer formation (38, 39, 82–84). This “inside-out” signaling complex is then engaged by the Vγ9Vδ2 TCR. Cryo-EM structures revealed a “plier-like gripping” mechanism in which BTN2A1 directly contacts Vγ9 laterally while BTN3A2 interacts apically with Vδ2 (40, 41). BTN3A1 also suppresses αβ T cell activation, while CD277-specific antibodies restore αβ T activity and elicit BTN2A1-dependent γδ T cytotoxicity (90). In contrast, BTNL3 and BTNL8 are bona fide transmembrane ligands that directly bind the Vγ4+ TCR via germline-encoded HV4 motifs (see next paragraph)—a mechanistically distinct mode of recognition from BTN3A1-mediated phosphoantigen sensing.

**Table 3:** Key ligands and their recognition mechanisms in γδ T cell activation.

Ligands	Functions	Citations	Evidence level
BTN3A1 (CD277)	Intracellular phosphoantigen SENSOR (not a direct TCR ligand); B30.2 domain binds PAg → conformational change → forms surface BTN3A1–BTN2A1 heterotetramer engaged by Vγ9Vδ2 TCR (inside-out signaling)	(82, 83)	Tier I (B30.2–PAg binding, structural); Tier II (Vγ9Vδ2 activation); Tier III (BTN3A1-KO mice lose PAg response)
BTN2A1	B30.2 domain interacts with BTN3A1-B30.2 upon PAg binding, essential for Vγ9Vδ2 TCR activation; IgV domain binds Vγ9 laterally	(38–41, 84)	Tier I (BTN2A1–BTN3A1 B30.2 heterocomplex by cryo-EM; IgV–Vγ9 binding by SPR/cryo-EM); Tier III (Btn2a1-KO abolishes Vγ9Vδ2 response)
BTN3A2	Interacts apically with Vδ2 in BTN multimers; required with BTN3A3 for BTN3A1-mediated PAg response; supports trafficking of V-domain-deleted BTN3A1	(40, 41, 84)	Tier I (BTN3A2 apo-Vδ2 contact in cryo-EM multimer); Tier II (BTN3A1 redistribution requires BTN3A2/3 co-expression)
BTN3A3	Required with BTN3A2 for BTN3A1-mediated PAg response; JM region supports functional complex formation and alters cellular distribution	(84)	Tier II (compensatory trafficking support; cannot substitute for BTN3A1 in PAg sensing)
BTNL3	Directly binds human Vγ4+ TCRs via IgV domain; interaction is enthalpically driven, involving germline-encoded Vγ4 regions (CDR2γ, HV4γ)	(85, 86)	Tier I (BTNL3 IgV–Vγ4 Kd ~15–25 μM by SPR/ITC; structural basis resolved); Tier II (colonic Vγ4+ TCR-dependent responses)
MR1	MHC-like molecule recognized by Vγ8Vδ3 TCR; structure of TCR–CD3 complex shows considerable conformational heterogeneity	(87)	—

PAg-mediated activation requires the PAg-sensing B30.2 domain and the TCR-engaging IgV domain on different BTN3A chains within a homo- or heteromer (84). BTN3A1 is essential; BTN3A2 and BTN3A3 cannot compensate in its absence (84). The juxtamembrane regions control trafficking and conformation of BTN3A complexes (84).

BTNL3 directly binds human Vγ4+ TCRs via germline-encoded Vγ4 regions (Kd ~15–25 μM), an enthalpically driven interaction where CDR3 mutations had negligible effects (85). This reveals two discrete binding modalities: non-clonotypic superantigen-like interactions via germline-encoded Vγ regions, and CDR3-dependent antibody-like interactions for clonally restricted antigens (85). In mouse gut, Btnl1 and Btnl6 jointly induce Vγ7+ IEL responses; in human gut, BTNL3 and BTNL8 induce colonic Vγ4+ cell responses (5). Combinatorial diversity of BTNL proteins selects and maintains signature intraepithelial γδ T cell compartments (91). Skint1 engages epidermal Vγ5Vδ1 TCRs for ‘normality sensing’ (6, 62, 92).

BTNL responsiveness is encoded by germline-encoded HV4 motifs within TCR Vγ chains. Vγ7 gene segment usage is the sole constant property of Btnl1-dependent IELs (3). The motifs responsible for BTNL recognition map to the HV4 region: Vγ4HV4 (DGKM motif) substitution with Vγ2HV4 (YANL motif) abrogates BTNL3 + 8 responsiveness (3). Replacement of Vγ7HV4 with human Vγ4HV4 confers responsiveness to human BTNL3 + 8, demonstrating evolutionarily conserved, functionally interchangeable motifs (3). γδ TCRs intrinsically combine innate and adaptive immunity using germline-encoded HV4 loops for non-clonal BTNL recognition and CDR3 loops for clone-specific recognition (3).

### Recognition of MHC class I-like molecules

IN HUMANS, Vδ1+ γδ T cells recognize CD1d presenting self-lipids like sulfatide, with the δ chain docking at a tilted angle and CDR3δ loop residues contacting sulfatide (1, 2). γδ TCRs can recognize CD1d in a lipid-independent manner (93). Atypical sideways recognition of CD1a by autoreactive Vδ1+ γδ TCRs has been demonstrated (94). Human γδ T cells exhibit autoreactivity to MR1 with a unique binding mode underneath the antigen-binding cleft (95). A human Vγ4Vδ5 TCR directly binds EPCR in an antibody-like, lipid-independent manner via CDR3γ and CDR3δ loops (85, 96). Classical murine studies established T10/T22 as non-classical MHC class Ib ligands for γδ TCRs (97), providing the foundational structural framework for understanding γδ TCR recognition of MHC-like molecules.

### Recognition of intracellular self-proteins and stress antigens

A new paradigm has emerged: γδ T cells recognize ectopically expressed intracellular self-proteins under stress, representing DAMP sensing (98). A screen of ~21, 000 human proteins identified nucleolin (NCL) as a Vδ1 TCR ligand and TGM1 for Vδ2 TCR, both upregulated on tumor cells and upon stress (98). The Vγ9Vδ1 TCR recognizes EphA2 upregulated upon AMPK-dependent metabolic reprogramming (99). NKG2D detects stress-induced ligands MICA/B and ULBP; tumor hypoxia represses NKG2D via PKA (100), while NKG2D-mediated detection of stressed hepatocytes is essential for NASH initiation (101). BTLA negatively regulates Vγ9Vδ2 T cell proliferation (102, 103). CD16 (FcγRIIIa) on HCMV-induced γδ T cells enables ADCC (104). NKp30 on Vδ1 T cells induces chemokines suppressing HIV-1 (105). KIR3DL3 has emerged as a novel inhibitory receptor recognizing HHLA2, enriched on γδ T cells in mucosal tissues and tumors (106).

γδ T cells directly recognize microbial antigens. Phycoerythrin (PE) is recognized directly by γδ TCRs without processing (KD 2.7 μM), with CDR3 regions essential for recognition (107). Antigen specificity for PE, but not specific TCR sequences, is conserved across species (107). Recent cryo-EM structures of a Vδ1Vγ5 TCR bound to PE revealed convergent use of apical aromatic residues shared with antibody–PE complexes, demonstrating that γδ TCRs can directly recognize foreign protein antigens in an antibody-like manner (108). These antibody-like and polyspecific recognition modes are illustrated in the right panel of **Figure 3**.

### Signaling mechanisms in humans

The ‘inside-out’ PAg sensing mechanism involves intracellular PAg binding to BTN3A1 B30.2 domain, inducing conformational change promoting BTN2A1-BTN3A heterotetramer formation engaging Vγ9Vδ2 TCR (39, 40, 84). Pure resting Vγ9Vδ2 T cells can self-activate through exogenous PAg involving their own TCR, BTN3A1, and BTN2A1, with ABCA-1 implicated in PAg internalization (109). ICT01, an anti-BTN3A antibody, activates Vγ9Vδ2 T cells independent of PAg (36).

### Signaling mechanisms in mice

PE immunization reveals antigen-driven IL-17 production: within 24 hours, PE-specific γδ T cells transition to activated state, with RORγt expression and IL-17A/F production by 48–60 hours (107). Antigen recognition induces IL-1 and IL-23 receptor expression, with continued calcineurin-NFAT signaling requirement (107).

### Integrative summary of antigen recognition and signaling

Transcriptomic analyses reveal a continuous ‘innateness gradient’ with γδ T cells at the innate pole, characterized by pre-formed effector mRNA enabling rapid responses (110). Innateness is associated with decreased ribosomal gene expression and impaired proliferation, revealing a trade-off (110).

In summary, γδ TCRs employ two discrete binding modalities: germline-encoded HV4 loops for non-clonal BTNL recognition and CDR3 loops for clone-specific recognition (85). Evolutionarily conserved HV4 motifs are the molecular determinants of BTNL/Btnl responsiveness (3). γδ TCR–CD3 complex assembly is Vγ-dependent with monomeric and dimeric architectures, and cholesterol acts as an inhibitory regulator (89). BTNL heteromers are functional units required for selection and maintenance (91). For BTN3A-mediated PAg sensing, a division of labor exists where the PAg-sensing B30.2 and TCR-engaging IgV domains must reside on different BTN3A chains (84). Pure resting Vγ9Vδ2 T cells undergo self-activation via ABCA-1-mediated PAg internalization (109). Ectopically expressed intracellular self-proteins represent a DAMP-like recognition strategy (98). KIR3DL3 is a novel immune checkpoint recognizing HHLA2 (106). Furthermore, γδ TCR polyspecificity enables cross-recognition of structurally distinct self-, microbial-, and viral-derived antigens (111), expanding the paradigm beyond single-ligand specificity.

## Tissue-resident γδ T cells and barrier immunity

Defining tissue residency — evidence criteria. Throughout this section, we apply a strict operational definition of tissue residency that distinguishes it from simple tissue enrichment or isolated CD69 expression. Genuine residency requires concordant evidence from at least two of the following four criteria: (i) long-term retention (>4 weeks) without blood-borne replenishment, demonstrated by parabiosis or fate-mapping (e.g., Kaede, tdTomato); (ii) local self-renewal via *in situ* proliferation (Ki-67+, BrdU retention); (iii) co-resident marker co-expression (CD69 together with CD103, PD-1, and/or TRM-associated transcription factors Runx3, Bhlhe40, Hobit); and (iv) downregulated egression program (low S1PR1, KLF2, TCF7). Simple tissue enrichment — increased frequency relative to blood without retention evidence — and isolated CD69 expression without other residency markers are explicitly labeled as provisional or incomplete evidence throughout. By these criteria, well-established γδ tissue-resident populations include mouse Vγ5+Vδ1+ DETCs, Vγ7+ intestinal IELs, and dermal Vγ4+ γδT17; and human Vδ1+ liver, breast, and epidermal subsets. Populations reported as ‘tissue-resident’ on the basis of enrichment alone (e.g., certain lung Vγ6+ and adipose PLZF+ γδ T cells) are noted as candidate or context-dependent residents pending further lineage-tracing evidence. The chemokine- and integrin-driven homing and retention mechanisms underlying this residency are summarized in the left panel of **Figure 4**.

**Figure 4:**
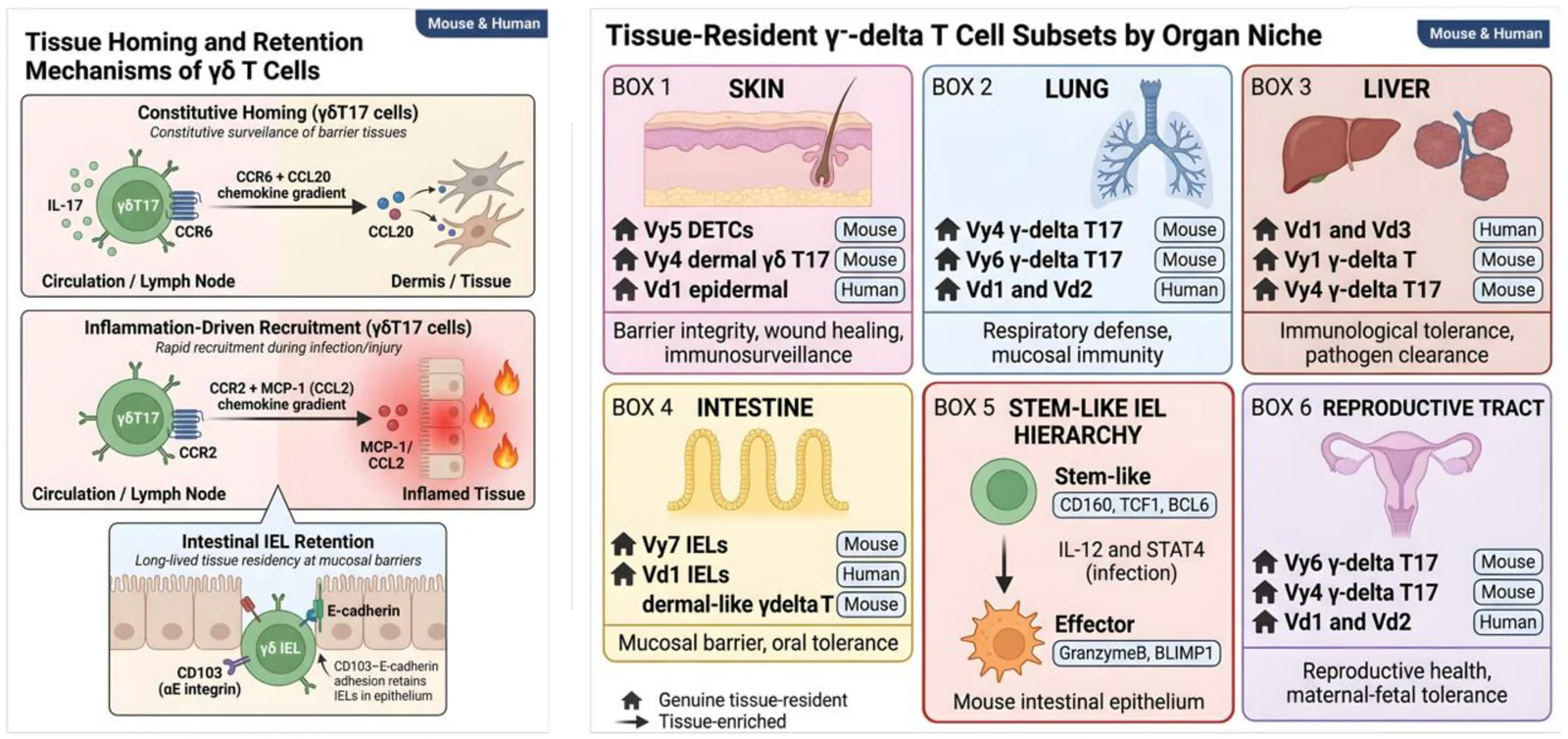
Tissue-resident γδ T cells: homing, retention, and niche specialization composite figure with two panels. Panel A (Homing and Retention Mechanisms) summarizes chemokine- and integrin-mediated trafficking: constitutive CCR6 -CCL20 recruitment of γδT17 cells to barrier tissues, inflammatory CCR2 -MCP-1 recruitment, and CD103 -E-cadherin -mediated retention of intraepithelial γδ T cells (IELs). Panel B (Niche-Specialized Subsets by Organ) organizes tissue-resident γδ T cell subsets into a six-box grid according to organ niche -- Skin (Vγ5+ DETCs, Vγ4+ dermal γδT17; human Vδ1+ epidermal), Lung (Vγ4+ and Vγ6+ γδT17; human Vδ1+/Vδ2+), Liver (Vδ1+/Vδ3+ human; Vγ1+/Vγ4+ mouse), Intestine (Vγ7+ IELs, Vδ1+ IELs), Stem-like IEL Hierarchy (130), and Reproductive Tract (Vγ6+/Vγ4+ γδT17; human Vδ1+/Vδ2+). The highlighted Stem-like IEL Hierarchy box depicts the differentiation flow from stem-like CD160+TCF1+BCL6+ progenitors to IL-12/STAT4-driven GranzymeB+BLIMP1+ effectors during infection. House and arrow icons distinguish genuine tissue residency (CD69+CD103+, long-lived, parabiosis-confirmed) from tissue enrichment (recruited, transient). Species banners identify mouse versus human content per box.

Tissue-resident γδ T cells are preprogrammed during thymic development for rapid responses to stress signals, microbial products, and dietary components at epithelial surfaces (7, 50). The literature on tissue residency is dominated by mouse studies (Vγ5+ DETCs, Vγ7+ IELs); human correlates (Vδ1+ liver/breast, Vδ2+ blood) are partially overlapping but use a different nomenclature and are not directly equivalent. Below, mouse and human findings are presented in separate species-specific subsections, with species and nomenclature specified accordingly. Cross-organ single-cell profiling reveals site-adapted subsets with transcriptional homogeneity yet epigenetic hallmarks of functional diversity (7). Comprehensive profiling across seven organs identified eight major γδ T cell subsets with distinct tissue distributions and functional specializations (7). Parabiosis experiments uncovered cellular states associated with cytotoxicity, innate-like IFN-γ production, and tissue repair that display residency hallmarks, revealing a shared core residency program across lymphocyte lineages (7).

Epithelial tissues shape local γδ T cell compartments through organ-specific Btnl molecules. Skint1, expressed by thymic epithelial cells and suprabasal keratinocytes, drives selective maturation of Vγ5+ DETC progenitors, with >90% ablation in Skint1 mutant mice (5). In the intestine, Btnl1 expressed by post-mitotic villus enterocytes selectively promotes Vγ7+ IEL maturation, with deficiency causing ~90% depletion (5). This selection is extrathymic, does not require microbial/food antigens, and acts in trans from non-hematopoietic cells (5). Btnl1 and Btnl6 jointly induce TCR-dependent responses (5). Human BTNL3 and BTNL8 induce selective TCR-dependent responses of colonic Vγ4+ cells (5). Niche-specialized tissue-resident subsets across organs are summarized in the right panel of **Figure 4**.

γδT17 cells are critical mediators of tissue regeneration. In bone defects, IL-17A was significantly upregulated, and regeneration was markedly impaired in Il17a−/− mice (27). The cellular source was predominantly Vγ6+ γδ T cells (~70% of γδ T cells in repair tissue, >80% of IL-17A+ cells expressing Vγ6) (27). IL-17A-responsive cells were PDGFRα+Sca-1+ mesenchymal stem cells that upregulated IL-17RA after injury (27).

Gut-to-lung migration of γδT17 cells contributes to sepsis-induced ALI. In a CLP model, γδ T cells were the dominant IL-17A source in the lung within 24 hours (112). Kaede-transgenic lineage tracing demonstrated that a substantial proportion of IL-17A+ γδ T cells in the septic lung originated from the small intestine (112). Migration was driven by Wnt/β-catenin signaling in alveolar macrophages (112). In obesity-associated colorectal carcinogenesis, IL-6-polarized macrophages produce CCL-20 recruiting CCR6+ γδ T cells (113). Myeloid-specific IL-6Rα deletion strongly ameliorated CAC (113).

γδT17 cells dynamically switch chemokine receptors: resting cells use CCR6 for dermal surveillance; upon activation, IRF4- and BATF-dependent CCR6 downregulation enables CCR2-driven migration to inflamed tissues (114). CCR6 directs homeostatic dermal recruitment; CCR2 is critically required for recruitment to inflamed tissues (114).

The ocular surface harbors C. mastitidis driving protective IL-17 from Vγ4+ γδ T cells in a TCR-dependent, CD1d- and IL-1-dependent manner (115). Colonization conferred γδ T cell- and IL-17-dependent protection against C. albicans and P. aeruginosa (115). In tobacco smoke-induced COPD, clonally expanded Vγ6+Vδ4+ γδT17 cells protect against lung injury (116). Tcrd−/− mice exhibited worsened smoke-induced lung injury, whereas adoptive transfer of γδT17 cells restored tissue protection (116).

In healthy human breast, Vδ1+ γδ T cells showing candidate tissue-resident phenotype (co-expressing NKG2D and CD69) are skewed toward cytolysis and IFN-γ production (117); formal parabiosis/fate-mapping evidence in humans is lacking, so residency assignment is provisional. In TNBC, Vδ1+ T cell representation correlates with survival (117). Iron homeostasis profoundly influences γδ T cells: in HFE-related hemochromatosis, Vδ2+ cells are depleted via chronic tonic TCR stimulation driving exhaustion (118).

## Skin-resident γδ T cells

### Murine skin γδ T cells: DETCs and dermal γδT17 cells

Vγ5Vδ1 DETCs reside in epidermis while dermal γδ T cells produce IL-17A (29). DETCs contribute to wound healing through CD100–plexin B2 interactions and keratinocyte growth factors, whereas dermal Vγ4+ γδ T cells are the primary initial source of Fgf9, which triggers Wnt activation in wound fibroblasts and promotes hair follicle neogenesis (see **Table 4**) (26).

**Table 4:** Barrier tissue-resident γδ T cells: skin and intestinal mechanisms (murine and human sections).

Tissue	Roles	Functions	Citations
Mouse — Skin			
Skin	Mouse DETCs (Vγ5Vδ1)	Reside in epidermis; essential for wound healing via production of keratinocyte growth factors (KGF/FGF7) and CD100–plexin B2 signaling; DO NOT produce Fgf9 (Fgf9 is sourced by dermal Vγ4+ cells — see below)	(26)
Skin	Mouse dermal γδ T cells (γδT17)	Predominantly produce IL-17A	(29)
Skin	Mouse dermal Vγ4+ γδ T cells	Primary initial source of Fgf9 during wound healing; expression increases before hair follicle placode formation	(26)
Skin	Fgf9	Triggers Wnt activation in wound fibroblasts; promotes hair follicle neogenesis; neutralizing antibody reduces WIHN, overexpression increases neogenic follicles	(26)
Skin	CD100 receptor (on DETCs)	Interacts with plexin B2 on keratinocytes to regulate γδ T cell activation and morphological changes for wound repair	(119)
Skin	CD100-plexin B2 binding	Triggers ERK phosphorylation and cofilin dephosphorylation, leading to actin reorganization, dendrite retraction, and cellular rounding in DETCs	(25)
Skin	Plexin B2 (on keratinocytes)	Upregulated within 18 hours of wounding; CD100 deficiency delays wound closure, phenocopying γδ T cell-deficient mice	(25)
Human — Skin			
Skin	Human Vδ1+ γδ T cells	Predominate in epidermis and dermis	(120)
Skin	Human Vδ2+ γδ T cells	Enriched in subcutaneous fat	(120)
Skin	Human dermal γδ T cells	Marked paucity compared to mouse; cluster with αβ T cells in perivascular pockets, suggesting transit between skin and blood	(26)
Skin	CD69 (on human skin γδ T cells)	Controls AhR-dependent IL-22 secretion by regulating L-tryptophan uptake via LAT1-CD98 complex; contributes to psoriatic inflammation	(77)
Skin	CD69-LAT1-AhR-IL-22 axis (human dermal)	CD69 stabilizes LAT1 to control L-tryptophan uptake, regulating AhR-dependent IL-22 secretion; critical for psoriasiform inflammation	(77)
Mouse — Intestine			
Intestinal	γδ IELs (homeostatic migration)	Migrate between epithelial layer and basement membrane, covering ~950 µm²/h in SPF mice; behavior directed by commensal bacteria	(131)
Intestinal	γδ IELs (germ-free/antibiotic)	Reduced coverage (~650 µm²/h) with altered vertical displacement	(131)
Intestinal	SFB mono-association	Rescues IEL positioning and dynamics	(131)
Intestinal	CD103 (αE integrin)	Limits pathogen translocation via γδ IEL retention and function	(12)
Intestinal	GPR55	Negatively regulates γδ IEL migration dynamics; signals through Gα13-Rho-ERM upon LPI binding	(132)
Intestinal	GPR55 deficiency	Faster migration, more epithelial interactions (35% vs 28% close to epithelial cells), increased γδ IEL frequencies in small intestine	(132)
Intestinal	TCRγδ+LAP+ regulatory subset	~20% of γδ T cells in lamina propria/PPs; express TGF-β1, GARP; derive from thymic CD27+IFN-γ+CCR9+α4β7+ γδT1 cells; regulated by ATF-3	(134)
Intestinal	CCR9 and β7 integrin	Critical for intestinal localization of LAP+ γδ T cells	(134)
Intestinal	GPR55 (homing/egress)	Negatively regulates T cell homing to small intestine and γδ T cell egress from Peyer’s patches	(132)
Intestinal	γδ IELs (T. gondii defense)	Tcrd KO increases parasite translocation; CD103 deficiency reduces invasive parasites by 63%	(12)
Intestinal	γδ IELs (S. typhimurium defense)	Tcrd KO increases bacterial translocation within 15 min; CD103 KO reduces invasion; γδ IELs localize to infected epithelial cells and lateral intercellular space	(12)
Intestinal	Occludin	Required for protective γδ T cell migration during S. typhimurium infection; deficiency causes migration defects and increased pathogen invasion	(12)
Human — Intestine			
Intestinal	CD103+Vγ4+ colonic γδ T cells	Distinct transcriptomic signature (TIGIT, KIR2DL4, CCR9, etc.); responds to BTNL3+BTNL8 but not PMA/ionomycin	(31)
Intestinal	Vδ2+ circulating subset	Responds to HMBPP via BTN3A1/BTN2A1-mediated TCR activation	(133)

At steady state, DETCs form immunological synapses at keratinocyte tight junctions via Vγ5 TCR recognition of Skint1, a BTNL protein expressed by differentiated suprabasal keratinocytes (6, 92). These TCR–Skint1 interactions are continuously required; Skint1 blockade causes barrier defects, inflammation, and impaired DETC-mediated protection against UVR-induced DNA damage (92). This “normality sensing” is critical for epidermal barrier integrity, independent of DETC survival (92).

Dermal γδ T cells are the major IL-17 producers in skin inflammation. In imiquimod-induced psoriasis, IL-23 stimulates dermal γδ T cells to produce IL-17, driving epidermal hyperplasia (29). IL-23 produced by CD301b+ dDCs is essential for dermal γδ T cell proliferation and IL-17A production during C. albicans infection (120). Development and maintenance of dermal γδT17 cells require oxysterol sensing through GPR183 (76). Genetic ablation of Gpr183 and Ccr6 causes severe depletion; dietary cholesterol exacerbates psoriatic inflammation via GPR183 (76). Sox13 mutation causes selective Vγ4+ γδT17 deficiency and protection from psoriasis, linking Vγ4+ γδT17 cell-derived IL-17 to neutrophil recruitment (68). Metabolic reprogramming of γδT17 cells under psoriatic conditions involves a switch toward aerobic glycolysis and ACC1-mediated *de novo* lipogenesis, supporting IL-17A production (121). BTLA is downregulated in RORγt+ cells, limiting IL-7 responsiveness and IL-17/TNF production; BTLA-deficient mice show enhanced psoriasis susceptibility (72).

In S. aureus epicutaneous exposure, dermal γδ T cells are the predominant IL-17A-producing cells driving IL-36R/MyD88-dependent inflammation (122). PSMα peptides trigger keratinocyte alarmin release (IL-1α, IL-36α) via MyD88, with both IL-1R and IL-36R required for IL-17 induction (123). IL-17A/F are critical effectors driving pathology without affecting bacterial clearance (123). This IL-36R/MyD88-dependent pathway is anatomically compartmentalized: epicutaneous exposure triggers IL-36α-driven inflammation, while intradermal infection relies on IL-1β for host defense (122).

Skin-resident γδ T cells engage in critical neuroimmune interactions. Nociceptive sensory neurons drive IL-23 production from CD301b+ dDCs, leading to IL-17A production from dermal γδ T cells during C. albicans infection (120). CGRP directly activates γδ T cells in rosacea (125). TRPA1 nociceptor activation promotes systemic skin regeneration through an IL-23–dendritic cell–γδ T cell–IL-17 cascade (126). A GD3 epidermal γδ T cell subset produces IL-3, lowering sensory neuronal activation threshold for allergens via STAT5, with numbers regulated by commensal microbes (127).

### Fgf9-mediated hair follicle neogenesis in mice

Dermal Vγ4+ γδ T cells are the primary initial Fgf9 source during wound healing, triggering regional fibroblast Wnt2a expression and Wnt activation (26). This initiates a feedback amplification loop where Wnt-activated fibroblasts upregulate Fgf9 (26). Tcrd−/− mice exhibit >60% reduction in WIHN (26). Fgf9 from γδ T cells induces Wnt activation in fibroblasts, which upregulate Fgf9 expression (26). Exogenous Fgf9 partially restores WIHN in Tcrd−/− mice (26).

### γδ T cell-mediated IgE responses in carcinogenesis in mice

Vγ5+ DETCs orchestrate tumor-protective IgE responses following epithelial DNA damage, shaping an autoreactive IgE repertoire that protects against epithelial carcinogenesis via FcϵRI (128). Tcrd−/− mice exhibit impaired IgE+ plasma cell responses without affecting IgG1+ responses (128). DMBA-induced IgE is autoreactive, binding damaged epithelial cells; this autoreactivity is absent in Tcrd−/− mice (128).

### γδ T cells in skin aging in mice

Dermal γδ T cells are central drivers of skin inflammaging, becoming polarized toward IL-17 with age (129). *In vivo* IL-17 blockade reduces proinflammatory state and delays age-related traits including epidermal thinning, barrier dysfunction, and reduced hair follicle regeneration (129).

### Human skin γδ T cells in homeostasis and disease

In humans, Vδ1+ cells predominate in epidermis/dermis and Vδ2+ cells in subcutaneous fat (119). Human skin γδ T cells express CD69, controlling AhR-dependent IL-22 via LAT1-CD98-mediated L-tryptophan uptake, contributing to psoriatic inflammation (77). In psoriatic patients, CD69+ Vγ9+ γδ T cell frequency correlates with PASI (77) (see **Table 4**).

Human skin γδ T cell subset compartmentalization has clinical relevance: Vδ1 lymphomas arise from epidermis/dermis, Vδ2 lymphomas from subcutaneous fat, with Vδ2 lymphomas associated with more aggressive features and worse prognosis (119). In Merkel cell carcinoma, pre-existing Vδ1 γδ T cells are strongly associated with ICB response, converging functionally with CD8 Trm cells (124).

## Intestinal γδ T cells

### Mouse intestinal γδ IELs: mechanisms of homeostasis and defense

Intestinal γδ IELs exhibit microbiota-dependent migration, covering ~950 µm² of epithelial surface per hour in SPF mice versus ~650 µm² in germ-free mice (131). They limit pathogen translocation via CD103-dependent retention and function (12) (see **Table 4**). GPR55 negatively regulates γδ IEL migration dynamics (132). A regulatory LAP-expressing subset resides in intestinal lamina propria and Peyer’s patches, inducing CD4+Foxp3+ Tregs in an MHC-II- and LAP-dependent manner (134). Recent work has identified stem-like CD160+TCF1+BCL6+ intraepithelial γδ T cells that differentiate into granzymeB+BLIMP1+ effector cells during infection via IL-12/STAT4–BLIMP1 signaling (130), revealing hierarchical tissue-adapted programming within the IEL compartment.

Intravital microscopy reveals γδ IEL innate defense: within 15 minutes of Salmonella challenge, Tcrd KO mice show increased bacterial translocation (12). γδ IELs rapidly localize to infected epithelial cells via occludin-dependent migration (12). Infection induces “flossing”—serpentine movement with increased intercellular migration doubling within 2 hours (131). The metabolic switch, movement, and immune response require pathogen sensing by epithelial cells via Myd88; epithelial-specific Myd88 deletion induces “germ-free-like” dynamics (131).

In mice, IL-17-producing γδ T cells maintain barrier integrity by regulating occludin (133). Dysregulation of γδ IELs precedes Crohn’s disease-like ileitis, with reduced signaling through the epithelial HNF4γ/BTNL axis (134) (these findings derive from murine models of Crohn’s-like ileitis and remain to be confirmed in human Crohn’s disease).

Intestinal γδ T cells sense dietary components, regulating carbohydrate digestion and absorption machinery by controlling IL-22 from ILC3s (137). Commensal microbiota influence γδ T cell function: dectin-1 signaling promotes stress responses via IL-17 (138). Gut-derived γδT17 cells migrate to lung during sepsis (112) and to liver in cholestatic disease, driving fibrosis (139). The gut-brain axis involves intestinal γδ T cells: antibiotic-induced dysbiosis reduces IL-17+ γδ T cell trafficking to leptomeninges, protecting against ischemic stroke (140).

### Human intestinal γδ T cells in IBD and celiac disease

In human IBD, the CD103+Vγ4+ subset is disproportionately diminished; restoration associates with sustained remission (31). In UC, six transcriptionally distinct γδ T cell clusters are identified, with active disease marked by reduction in CD103+Vγ4Vδ1+ γδ IELs and emergence of memory-like (TCF-1+PD-1+) and effector-like (T-bet+GzmB+) subsets (32). TCR repertoire analysis reveals these represent infiltrating populations rather than rewired γδ IELs (32). Inflamed UC tissue shows downregulation of BTNL3/BTNL8 and upregulation of BTN3A1/BTN3A3 (32). Clinical improvement with anti-TNF or anti-integrin therapies was associated with recovery of γδ IELs; nonresponders retained active disease features (32). Human colonic γδ T cells include a CD103+Vγ4+ subset responding strongly to BTNL3+BTNL8-mediated TCR engagement (31). γδ IELs contribute to pathological epithelial cell shedding in IBD via CD103-mediated granzyme release (132).

In human studies of celiac disease, gluten-induced inflammation permanently depletes Vγ4+/Vδ1+ IELs with innate cytolytic properties and BTNL3/BTNL8 specificity, replaced by IFN-γ-producing Vδ1+ IELs (135). Gluten exclusion is insufficient to reconstitute the physiological subset, demonstrating permanent, site-specific remodeling (135). Single-cell multiomics identifies a TNM1 immunoregulatory population and an innate-like NCR+ population permanently lost from CeD mucosa, with persistent TNM2 expansion constituting “permanent scarring” (136).

## Lung-resident γδ T cells in mice

Lung γδ T cells provide early protection against respiratory infections. During influenza, host-derived lipids activate lung γδ T cells via CD1d+ B-1a cells to produce IL-17A (141). Ketogenic diet feeding expands protective, IL-17-competent γδ T cells in the lung, enhancing antiviral resistance through metabolic adaptation involving fatty acid oxidation (142). Commensal microbiota promote lung cancer by activating Vγ6+ γδ T cells through Myd88-dependent IL-1β and IL-23 (143). In neonatal influenza, IL-17A-producing γδ T cells promote protective tissue repair via IL-33-dependent ILC2 and Treg accumulation (13). In COPD, clonally expanded γδT17 cells represent a protective hallmark (116). In the PPE-induced emphysema model, lung-resident γδ T cells are the predominant IL-17A source, driven by IL-1β and IL-23, stimulating G-CSF-dependent differentiation of pathogenic Siglec-F+ neutrophils via JAK2/STAT3, PI3K-independent mTOR, and p38 MAPK pathways (144). Neutralizing G-CSF or inhibiting these pathways suppresses Siglec-F+ neutrophil development and alleviates symptoms (144).

Environmental chitin rapidly activates resident γδ T cells to produce IL-17A and mediate neutrophil recruitment, with ILC2s suppressing excessive activation (145). ST2+ Treg cells suppress IL-17-producing γδ T cells via IL-35, serving as a critical checkpoint restraining innate γδ T cell responses after mucosal injury (146).

## Adipose tissue-resident γδ T cells in mice

Adipose tissue harbors a highly enriched γδ T cell population (>90% host-endogenous in parabiosis assays, satisfying retention criterion) (28); however, formal demonstration of local self-renewal and TRM transcriptional programs is partial, so these cells are best characterized as candidate tissue-resident. Two populations exist: CD3ϵhiCD27− PLZF+ cells producing IL-17A/TNF, and CD3ϵloCD27+ PLZF− cells producing IFN-γ (28). These cells control fat innervation and thermogenesis via the adipocyte IL-17RC axis (147). Cold exposure specifically induces IL-17F, not IL-17A, in brown adipose tissue; Il17f−/− mice exhibit impaired cold tolerance (147). IL-17F signals through adipocyte IL-17RC, driving TGFβ1 expression to promote sympathetic innervation (147). Adipocyte-specific Il17rc deletion impairs cold tolerance and causes obesity, glucose intolerance, and hepatic steatosis on a high-fat diet (147). Ketogenic diet expands metabolically protective γδ T cells in visceral adipose tissue that restrain inflammation (148). Rhythmic IL-17 production by γδ T cells maintains adipose *de novo* lipogenesis, linking circadian immune function to metabolic homeostasis (149). γδ T cell-stromal networks modulate matrix composition and vascularity in the foreign body response, with γδT17 cells activating fibroblast ECM remodeling programs (150).

## Liver-resident γδ T cells

### Human liver γδ T cells

### Murine liver γδ T cells in NASH, fibrosis, and cholestasis

In cholestatic liver disease, altered intestinal microbiota drive IL-17 production by intrahepatic γδ T cells, promoting hepatic fibrosis and inflammation (139). Liver-resident γδ T cells have been identified as critical initiators of NASH progression. Under metabolic stress, hepatocyte cholesterol accumulation induces NKG2D ligands; tissue-resident γδ T cells are activated and secrete IL-17A, licensing hepatocytes to produce chemokines that recruit proinflammatory myeloid cells (101). NKG2D-deficient mice show striking reductions in liver fibrosis and hepatic tumor incidence in NASH models (101). scRNA-seq analysis identified a thymus-independent developmental pathway for liver-resident γδ T cells (152).

The human liver is enriched for γδ T cells; intrahepatic Vδ2- populations (predominantly Vδ1+) are clonally expanded and exhibit CD69+ tissue-resident-like effector phenotypes (151). As human lineage-tracing is not feasible, residency is inferred from co-expression of CD69/CD103 and lack of blood contamination, and is therefore classified as TIER II (cellular) evidence.

## Cross-talk with stromal cells and neurons

γδ T cells engage in bidirectional communication with stromal cells and neurons to maintain tissue homeostasis. In skin, DETCs interact with keratinocytes through CD100-plexin B2 and Skint1-dependent “normality sensing” (25, 92). In adipose tissue, γδ T cells regulate sympathetic innervation via IL-17RC signaling on adipocytes (147). Neuroimmune interactions are prominent: nociceptors drive IL-23 from dDCs to activate γδ T cells (120), CGRP directly activates γδ T cells in rosacea (125), and GD3 cells produce IL-3 to regulate neuronal allergen responsiveness via STAT5 (127). These tissue-specific stromal, metabolic, and neuroimmune cross-talk pathways are summarized in **Figure 5**.

**Figure 5:**
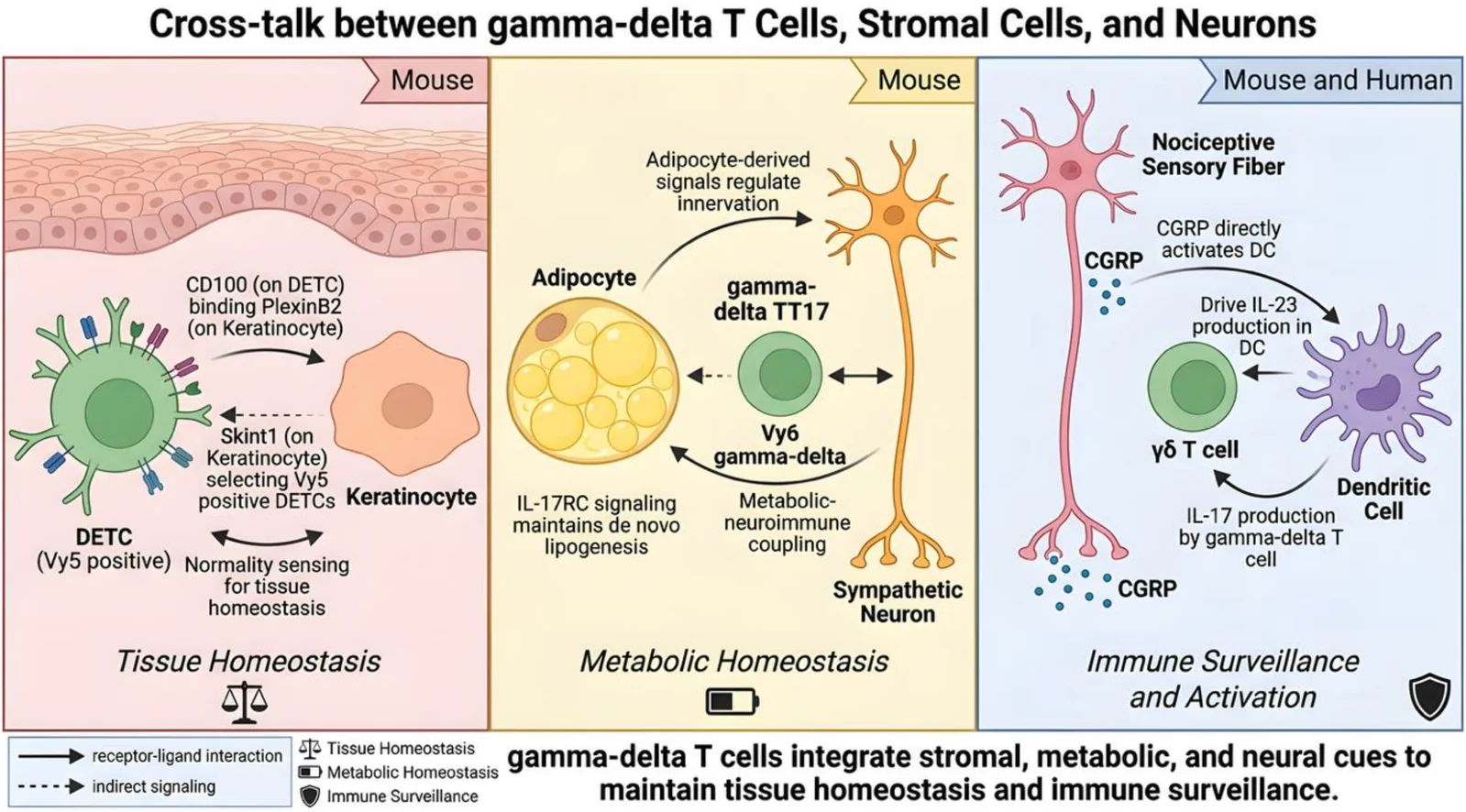
Cross-talk between γδ T cells, stromal cells, and neurons widescreen illustration highlighting multifaceted interactions between tissue-resident γδ T cells and non-hematopoietic/stromal/neural compartments across three contexts. Panel 1 (Skin, mouse): Dendritic epidermal T cells (DETCs, Vγ5+) engage keratinocytes through CD100 -PlexinB2 and are selected by Skint1, supporting normality sensing and tissue homeostasis. Panel 2 (Adipose tissue, mouse): Vγ6+ γδT17 cells maintain de novo lipogenesis in adipocytes via IL-17RC signaling, coupling metabolic and neuroimmune regulation with sympathetic innervation. Panel 3 (Neuroimmune, mouse and human): Nociceptive sensory fibers release CGRP that activates dendritic cells, which in turn produce IL-23 to drive γδ T cell IL-17 responses, completing a sensory-neuro-immune amplification loop. The bottom banner summarizes that γδ T cells integrate stromal, metabolic, and neural cues to maintain tissue homeostasis and immune surveillance. A balance-scale, battery, and shield icon triplet indicates Tissue Homeostasis, Metabolic Homeostasis, and Immune Surveillance, respectively.

Meningeal γδT17 T cells regulate anxiety-like behavior through IL-17a signaling in cortical glutamatergic neurons (153). A distinct fetal-derived Vγ6+ meningeal subset promotes short-term spatial memory through non-inflammatory IL-17 production, stimulating glial BDNF and enhancing hippocampal synaptic plasticity (154). In early-life neurodevelopment, elevated circulating γδ T cells correlate with brain damage in premature infants, implicating γδ T cell-derived IL-17a in perinatal white matter injury (155). In fibrosis, γδT17 cell-derived IL-17 activates ECM remodeling programs in fibroblasts and endothelial cells (150).

## γδ T cells in infection and host defense

γδ T cells serve as critical early responders against diverse pathogens. Unless otherwise noted, murine studies use Heilig–Wilson nomenclature and human studies use IMGT nomenclature. In human CMV, Vδ1+ cells undergo robust expansion, reaching 12% of T cells in CVID, with effector-memory phenotype and highly clonal repertoire (160). In human malaria, Vδ2+ cells kill P. falciparum merozoites via granulysin in a TCR-dependent, APC-independent manner (161). However, repeated exposure causes progressive Vδ2+ loss and dysfunction, with upregulation of immunoregulatory genes including HAVCR2 and BATF, associated with clinical tolerance (161). Single-cell transcriptomics reveals tolerogenic signatures across multiple immune subsets (162) (see **Table 5**).

**Table 5:** Antiviral defense mechanisms of γδ T cells (predominantly human: CMV, HIV, influenza, SARS-CoV-2).

Cells	Functions	Citations
Vδ2neg (Vδ1/Vδ3) γδ T cells	Long-term expansion during CMV reactivation after allogeneic stem cell transplantation; CMV-specific clonal proliferation	(16)
Vδ1 cells	Expand during CMV reactivation in HLA-haploidentical transplantation; enhanced cytotoxicity and degranulation against leukemia blasts (dual anti-viral and anti-leukemic potential)	(163)
CD16+ γδ T cells	Produce IFN-γ when incubated with IgG-opsonized virions	(104)
Vδ1+ products	Control CMV dissemination via LFA-1 co-stimulation, independent of the γδ TCR	(164)
Vδ1+ cells in CVID	CMV drives Vδ1+ expansion in common variable immunodeficiency	(165)
MCTS1 deficiency	Selectively impairs JAK2 translation and IL-23 response in Vδ2+ γδ T cells, revealing essential role in IFN-γ-dependent antimycobacterial immunity	(166)
Vγ9Vδ2 T cells (influenza)	Kill infected lung epithelial cells via NKG2D, perforin-granzyme B, TRAIL, and Fas-FasL pathways	(160)
H5N1 hemagglutinin	Directly activates γδ T cells	(167)
CD137/CD137L costimulation	CD137 induced on ~20% of Vγ9Vδ2 T cells; CD137L upregulated on 25.7% of infected MDMs; prevents exhaustion, enhances cytotoxicity, reduces viral titers, and improves survival	(168)
γδ T cell-derived IL-17A (neonatal)	Promotes tissue repair via IL-33-dependent ILC2 and Treg accumulation in neonatal influenza infection	(13)
γδT17 cells (lung)	Major early IL-17A source; activated by CD1d+ B-1a cell-presented endogenous lipids via γδTCR-mediated IRF4-dependent transcription; adoptive transfer rescues mice from lethal infection	(141)
Vδ2 subset (HIV)	Depleted via gp120 binding to CCR5/α4β7, activating p38-caspase-mediated cell death	(169)
Memory Vδ2 cells (HIV)	Skewed toward TEMRA phenotype in chronic infection; IL-17-producing γδ T cells correlate with fast progression	(170)
Gut-homing Vδ2 cells (HIV)	Acute HIV-1 upregulates Δ42PD1	(171)
Vδ1/Vδ2 inversion (SIV)	Occurs without direct infection	(172)
NKp30 on Vδ1 T cells (HIV)	Engagement induces CCL3/4/5 to suppress HIV-1 replication	(105)
Vδ2 γδ T cells (SARS-CoV-2)	Pronounced decrease correlating with disease severity; increased CD38 activation	(173)
VD1 γδ T cells (SARS-CoV-2)	Numbers remain stable; increased CD38 activation	(173)
Immature neutrophil-to-Vδ2 T-cell ratio	Predicts pneumonia (AUC = 0.9071) and hypoxia (AUC = 0.8908), suggesting strong mobilization into lungs	(173)

Activation modes framework. γδ T cells engage pathogens through four mechanistically distinct activation modes that often operate cooperatively during a single infection. (i) Direct TCR-mediated recognition — e.g., Vγ9Vδ2 TCR recognition of Plasmodium-derived phosphoantigens with granulysin-mediated killing of merozoites (156), and butyrophilin-gated sensing of microbial metabolites. (ii) Cytokine-driven activation — e.g., IL-1β/IL-23-driven IL-17A production in Listeria and helminth infection (18, 157), IL-18-driven IFN-γ from sentinel-macrophage-adjacent γδ T cells in bacterial lymph-node responses (158), and IL-12/IL-23 axes in antimycobacterial immunity (159). (iii) NK-receptor-mediated recognition — e.g., NKG2D recognition of stress-induced MICA/B/ULBP in influenza-infected cells (160), NKp30-driven CCL3/4/5 secretion in HIV (105), and KIR3DL3 recognition of HHLA2. (iv) Antibody-dependent activation — e.g., CD16 (FcγRIIIa)-mediated ADCC by CMV-induced Vδ2neg γδ T cells against IgG-opsonized virions (104). The pathogen-based sections below should be read through this mechanistic lens; most infections engage multiple modes simultaneously, and the dominant mode often varies with tissue compartment and pathogen load.

In antimycobacterial immunity, RORC mutations impair IFN-γ production by γδ T cells, causing candidiasis and mycobacteriosis (161). IL-12 and IL-23 axes play distinct cooperative roles: γδ T cells preferentially respond to IL-12, while NKT and MAIT cells respond to IL-23 (159). Persistent controlled Mtb infection elicits expansion of CD8+ γδ T cells with “memory inflation” features, expressing NK-cell-associated receptors and mounting robust CD16-mediated cytotoxic responses (162).

### Antiviral defense

During CMV reactivation after allo-HSCT, Vδ2neg γδ T cells undergo long-term clonal expansions (16, 17). CD16+ γδ T cells produce IFN-γ with IgG-opsonized virions (104). Adoptive transfer of Vδ1+ products controls CMV via LFA-1 co-stimulation (164). Against influenza, Vγ9Vδ2 T cells kill infected cells via NKG2D, perforin-granzyme B, TRAIL, and Fas-FasL (160). CD137 costimulation prevents exhaustion and enhances antiviral activity (168). In HIV, Vδ2 cells are depleted via gp120-induced p38-caspase-mediated cell death (169). NKp30 engagement on Vδ1 T cells induces CCL3/4/5 suppressing HIV-1 (105). In SARS-CoV-2, Vδ2 γδ T cells decrease correlating with disease severity; immature neutrophil-to-Vδ2 ratio predicts pneumonia (AUC = 0.9071) (173).

### Antibacterial defense

In mouse models, γδ T cells provide early protection against bacterial infection via rapid cytokine production and cytotoxicity. In Listeria, mucosal γδ T cells generate multifunctional memory cells producing IFN-γ and IL-17A (18). γδ IELs limit Salmonella translocation via CD103-mediated migration (12). Within lymph nodes, γδ T cells near sentinel macrophages receive IL-18 signals to secrete IFN-γ, limiting bacterial spread (158).

In humans, RORC mutations cause defective anti-mycobacterial IFN-γ production (161), with IL-12 and IL-23 both essential for optimal immunity (159).

### Antiparasitic defense

During murine malaria, γδ T cell frequency multiplies 30-fold in blood by 21 days post-infection, with clonally expanded Vδ6.3 γδ T cells producing M-CSF preventing parasitemic recurrence (174, 175). γδ T cells also initiate adaptive CD8+ T cell immunity during malaria by providing IL-4, synergizing with IFNγ to induce IL-12 from cDC1s (176). In helminth infection, γδ T cells are critical IL-17A sources, with chitinase-like proteins promoting expansion via IL-1β (157).

In humans, Vγ9Vδ2 T cells form immune synapses with iRBCs and lyse them via granulysin-mediated membrane damage, with P. falciparum cholesterol depletion enhancing granulysin susceptibility (14).

### Effector mechanisms

Direct cytotoxicity is mediated primarily through perforin-granzyme pathway and death receptor ligands (160). Live single-cell imaging reveals limited co-occurrence of granzyme B and perforin restrains serial killing (177). IFN-γ is critical for antiviral and antibacterial immunity (158). IL-17A is essential for neutrophil recruitment and tissue repair (13, 157, 178). IL-17-producing human Vγ9Vδ2 T cells differentiate with phosphoantigens and IL-1β/TGF-β/IL-6/IL-23, inducing CXCL8-mediated neutrophil migration (179).

### Memory-like responses

γδ T cells undergo clonal expansion and exhibit memory-like responses. Human Vδ1+ TCR repertoire becomes strongly focused on high-frequency clonotypes stably maintained over months, differentiating to effector phenotype while retaining proliferative capacity (180). CMV reactivation drives clonal proliferation of virus-reactive γδ TCR sequences (16). In malaria, clonally expanded Vδ6.3 cells with convergent CDR3 sequences emerge post-parasitemia resolution (174). Oral Listeria induces multifunctional memory γδ T cells with ~70% producing IL-17A and ~25% co-producing IFN-γ, exhibiting contextual specificity for the priming pathogen (18).

## γδ T cells in cancer: dual roles in tumor immunity

Evidence levels in oncology. To facilitate critical appraisal, mechanistic and clinical claims below are tagged with their evidence tier. Tier I (clinical) — data from interventional clinical trials (predominantly non-randomized phase I/II in this field). Tier II (human correlation) — observations from patient samples, ex vivo tumor analyses, or correlative biomarker studies (no causal intervention). Tier III (preclinical) — syngeneic mouse models, xenografts, or *in vitro* mechanistic studies. Where mouse and human data diverge, both species tags are shown. The predominance of Tier III evidence for mechanistic claims and Tier II evidence for clinical correlations—combined with the near-absence of randomized phase III data—should temper efficacy assertions (see Limitations section). The dual roles with evidence-level color-coding are visualized in **Figure 6**.

**Figure 6:**
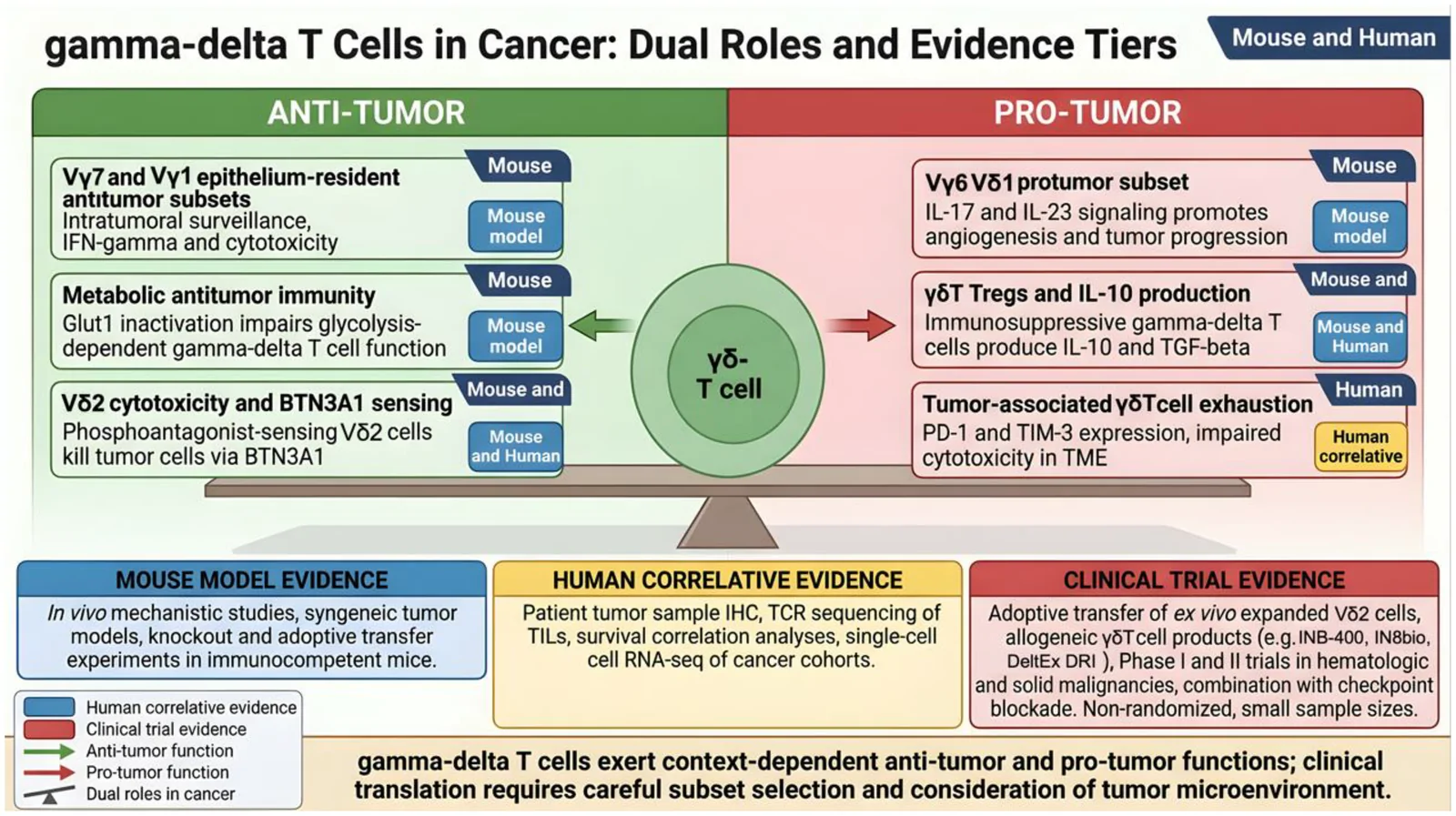
γδ T cells in cancer: dual roles and evidence tiers widescreen figure visualizing the context-dependent dual functions of γδ T cells in cancer with explicit evidence-level tagging. A central balance-beam motif contrasts anti-tumor (green, left) and pro-tumor (red, right) roles. Anti-tumor activities include Vγ7+/Vγ1+ epithelium-resident cytotoxic subsets, Glut1-dependent metabolic antitumor immunity, and Vδ2 -BTN3A1 phosphoantagonist-sensing cytotoxicity. Pro-tumor activities include the Vγ6Vδ1 subset promoting angiogenesis via IL-17/IL-23, IL-10 -producing γδ Tregs, and PD-1/TIM-3 -associated exhaustion in the tumor microenvironment. Each mechanistic box carries a color-coded evidence tag -- blue for mouse-model evidence, yellow for human correlative evidence, red for clinical trial evidence -- and a species banner (mouse, human, or mouse and human). A bottom strip summarizes the three evidence tiers and cautions that current clinical data are predominantly from non-randomized Phase I/II studies.

γδ T cells exhibit functional dichotomy in cancer, exerting both antitumor and pro-tumor functions shaped by subset and TME (see **Table 1**). In CRC, Vγ7+ and Vγ1+ cells with epithelium-resident phenotype (satisfying Runx3+ CD103+ criteria) exhibit cytotoxic, IFN-γ-producing anti-tumor profiles, while tumor-infiltrating Vγ6+Vδ1+ cells adopt PD-1+ IL-17-producing pro-tumorigenic phenotype (21) (in mice). DNMTis upregulate NKG2D ligands and death receptors, potentiating Vδ1-enriched cytolysis (186).

The IFN-γ/IL-17 dichotomy is underpinned by distinct metabolic programs imprinted during thymic development: γδIFN depend on glycolysis (Myc-driven), γδT17 engage OXPHOS (Nrf1-driven) (75). In TME, γδT17 cells exhibit high lipid uptake; glucose supplementation enhances γδIFN anti-tumor functions (75).

Dietary fatty acids modulate Vγ9Vδ2 T cell function: palmitic acid suppresses antitumor activity by disrupting glycolysis/OXPHOS and inducing pyroptosis via excessive IFN-γ, while oleic acid restores function (181). The STING pathway intrinsically regulates γδ T cell antitumor immunity via TBK1–Eomes–IFN-γ axis; TGF-β downregulates STING, and combining TGF-β inhibition with STING agonists augments antitumoral effects (182).

BTN3A1 is overexpressed in solid tumors, suppressing αβ T cell activation by binding N-glycosylated CD45 (90). Anti-BTN3A antibodies relieve suppression and trigger BTN2A1-dependent Vγ9Vδ2 cytotoxicity (90). BTNL2 suppresses antitumor immunity by engaging IL-17A-producing γδ T cells; blockade reduces tumor growth and establishes immune memory (24). Genome-wide CRISPR screens identify IRF1 and ZNF217 as positive/negative regulators of BTN3A, with metabolic crises upregulating BTN3A/BTN2A1 in AMPK-dependent manner (20).

In MSS CRC, Vδ1+ T cells undergo TCR repertoire reshaping with dysfunction driven by fibroblast TIGIT-NECTIN2 axis (183). In RCC, Vδ2− γδ T cells exhibit exhausted phenotype yet retain effector function and kill autologous tumor cells; their transcriptional signature predicts PD-1 blockade response (184). In HCC, sexual dimorphism in Treg infiltration suppresses γδ T cell activity via androgen-driven Ccl2-mediated Treg recruitment; Tregs suppress γδ T cells through hypoxia-induced S100a4-enriched EVs (185). Commensal microbiota drive lung adenocarcinoma via Vγ6+Vδ1+ γδT17 cells through Myd88-dependent IL-1β and IL-23 (143). In human NSCLC [IMGT nomenclature], Vδ1 T cells are the predominant intratumoral subset with CD103+ TRM phenotype and Tc1 program associated with survival (186). In AML, Vδ1+ DOT cells exhibit robust cytotoxicity via DNAM-1 ligands and NKp30 ligand B7-H6 (187).

### Anti-tumor functions

Vγ9Vδ2 T cells recognize phosphoantigens via BTN3A1/BTN2A1, triggering perforin-granzyme and death receptor killing (38, 82, 160). In ovarian cancer, TIL γδ T cells exhibit CD103+ tissue-resident-like phenotype correlated with beneficial outcomes (188) (human correlative evidence); formal residency criteria have not been applied. In NSCLC, Vδ1 T cells with resident/effector memory phenotypes are associated with improved survival (186). IL-36γ synergizes with TCR signaling to stimulate CD8+ T, NK, and γδ T cells, inhibiting tumor growth and reducing metastasis (189). In a non-randomized phase I trial (n=132), adoptive Vγ9Vδ2 T cell therapy demonstrated acceptable safety; an exploratory subgroup receiving ≥5 infusions exhibited prolonged survival, although this *post-hoc* observation requires confirmation in randomized trials (37). Engineered γδ T cells secreting anti-PD-1 antibodies show enhanced efficacy (190).

### Pro-tumor functions

In breast cancer, tumor-induced IL-1β-driven IL-17 from γδ T cells triggers G-CSF-dependent neutrophilia facilitating metastasis (23). 27-hydroxycholesterol promotes metastasis by increasing γδ T cells at metastatic sites (191). In PDA, γδ T cells constitute up to 75% of TILs and restrain αβ T cell activation via PD-L1 and Galectin-9; depletion yields ~10-fold increase in activated αβ T cells (22, 192). In skin carcinogenesis, activin A induces progressive loss of tumor-suppressive Vγ3+ DETCs (193).

### Prognostic biomarkers and checkpoint blockade

A dysfunctional Vγ9Vδ2 T cell phenotype in CLL predicts shorter time-to-first-treatment (194). Reduced CDR3δ repertoire diversity serves as diagnostic marker in lung carcinoma (195). In melanoma, intratumoral TRDV1 transcripts predict anti-PD-1 response (196). In AML, oligoclonal TRDV4/TRDV8 expansion are protective factors for complete remission, while TRDV5/TRDV6 expansion are risk factors for relapse (197).

In HLA class I-deficient cancers, γδ T cells mediate immunotherapy responses (19, 198). In MMR-d cancers with B2M inactivation, TRDV1 and TRDV3 are among most significantly upregulated genes, with intratumoral Vδ1 and Vδ3 subsets exhibiting high PD-1 and cytolytic molecules (19). PD-1 on Vδ1+ T cells defines a functional tissue-adapted state distinct from classical exhaustion (196). γδ T cell depletion significantly decreases PD-1 blockade efficacy (199).

## γδ T cells in autoimmunity and chronic inflammation

γδ T cells are critical mediators in autoimmune and chronic inflammatory diseases through rapid IL-17 production (see **Table 1**). In psoriasis, dermal γδ T cells are the dominant IL-17 source, driving epidermal hyperplasia via IL-23/IL-17 axis (29). Psoriatic γδT17 cells switch to aerobic glycolysis and ACC1-mediated *de novo* lipogenesis, with pharmacological ACC1 inhibition ameliorating inflammation (122). CD69 controls AhR-dependent IL-22 via LAT1-CD98 (77). Neonatal antibiotic dysbiosis increases IL-22-producing γδ T cells exacerbating psoriasis, an effect transmissible by cohousing (207). In skin aging, IL-17-skewed γδ T cells drive chronic inflammation, delayed by IL-17 blockade (130).

In rheumatoid arthritis, Vγ9Vδ2 T cells acquire APC characteristics presenting antigens to CD4+ T cells (200). In spondyloarthritis, RORγt-expressing innate-like T cells contribute to dysregulated IL-23/IL-17, with RORγt inhibition selectively targeting IL-17-producing populations while preserving IL-22 (201). In IL-1Ra-deficient mice, Vγ6+ CCR2+ γδ T cells are primary IL-17 producers in inflamed joints, recruited via CCL2 (33).

In MS and EAE, CD161high CCR6+ γδ T cells are enriched in CSF during active disease (202). IL-23-activated γδ T cells antagonize Treg-mediated suppression; Tcrd−/− mice are protected from EAE unless Tregs are depleted (30). In ischemic stroke, γδ T cell-derived IL-17A drives neutrophil recruitment (178). In TBI, Vγ4 cells secrete IFN-γ and IL-17 while Vγ1 cells secrete TGF-β; TCRδ−/− mice show reduced lesion volume and improved outcomes (203).

In IBD, Treg cells maintain homeostasis by suppressing γδ T cells (204). The ubiquitin ligase Itch targets RORγt for ubiquitination, inhibiting IL-17-mediated colitis (205). In celiac disease, Vγ4+ γδ T cells are permanently depleted (135). Regulatory LAP-expressing γδ T cells induce Tregs maintaining mucosal tolerance (206).

In obesity, adipose γδ T cells regulate Treg homeostasis and thermogenesis (28, 147). Rhythmic IL-17 maintains adipose *de novo* lipogenesis (149). Ketogenic diet expands metabolically protective γδ T cells (148).

In neuroinflammation, meningeal γδT17 T cells regulate anxiety via IL-17a signaling in cortical neurons (153). A fetal-derived Vγ6+ subset promotes short-term memory through IL-17-glial BDNF axis (154). Colonic γδ T cells modulate behavioral vulnerability to stress via dectin-1 signaling (138). In premature neonates, aberrant gut-microbiota-immune-brain axis with elevated γδ T cells is associated with brain damage (155).

Regulatory mechanisms include Aire inhibiting perinatal γδT17 generation (52), IL-33 activating Tregs to suppress γδ T cell responses via IL-35 (146), and LAP+ γδ T cells inducing Tregs (206). Selective RORγt targeting blocks pathogenic IL-17 while preserving IL-22 (201). Targeting ACC1-mediated lipogenesis selectively impairs pathogenic γδT17 cells (121).

## Therapeutic targeting and clinical translation

### Adoptive cell transfer

Vγ9Vδ2 T cells are expanded using aminobisphosphonates or synthetic pAgs, which inhibit the mevalonate pathway leading to IPP accumulation sensed by BTN3A1 (82, 83) (see **Table 6**). IL-15 enhances proliferation and antitumor functions (216). Vitamin C promotes proliferation, reduces apoptosis, and augments effector functions (217). A novel formula (NF) combining ZOL, IL-2, IL-15, and vitamin C significantly enhances expansion and effector functions (37). Vδ1 T cells recognize stress ligands on epithelial tumors; the DOT cell platform demonstrates broad AML cytotoxicity dependent on CD155/PVR and B7-H6, and controls CMV via LFA-1 (170, 193). In a non-randomized phase I trial, allogeneic γδ T cell therapy showed acceptable safety, with prolonged survival restricted to a *post-hoc* subgroup receiving ≥5 infusions; definitive efficacy awaits randomized confirmation (37). TCRdb2.0 incorporates ~700 million TCR sequences enabling systematic analysis (220). Dynamic monitoring of malignant γδ T cell clones enables MRD detection (222). The overall therapeutic landscape, encompassing adoptive cell therapy, *in vivo* activation, and engineering or combination strategies, is summarized in **Figure 7**.

**Table 6:** Adoptive cell transfer strategies for γδ T cells (preclinical models and early-phase clinical trials).

Strategies	Contents	Citations
Aminobisphosphonates (e.g., zoledronic acid)/Synthetic phosphoantigens	Inhibit mevalonate pathway, causing IPP accumulation sensed by BTN3A1 to expand Vγ9Vδ2 T cells	(82, 83)
IL-15	Enhances proliferation, activation, Th1 polarization, IFN-γ/TNF-α production, and cytotoxicity against Daudi and U266 cells	(207)
IL-15 plus IL-2	Boosts γδ T cell expansion from healthy donors and AML patients, strengthening antitumor properties	(207)
Vitamin C supplementation	Promotes proliferation, reduces apoptosis, improves cell cycle, augments cytokine production and metabolic fitness	(208)
Novel formula (ZOL + IL-2 + IL-15 + vitamin C)	Enhances Vγ9Vδ2 proliferation, differentiation, effector functions, mitochondrial mass, and cytotoxicity	(37)
Anti-γδ TCR antibodies	Yield sustained proliferative capacity and activity against lymphoid malignancies	(209)
Vδ1 T cells (NKp30 expression)	Recognize stress ligands; kill lymphoid leukemia cells resistant to Vγ9Vδ2-mediated lysis	(210)
Delta One T (DOT) cell platform	Broad AML cytotoxicity dependent on DNAM-1 ligands CD155/PVR and NKp30 ligand B7-H6	(187)
DOT cells (CMV control)	Control CMV infection via LFA-1 co-stimulation; protect mice against lethal murine CMV	(164)
Allogeneic γδ T cells (phase I trial)	Favorable safety and prolonged survival in late-stage liver/lung cancer patients	(37)
NF-expanded allogeneic Vγ9Vδ2 T cells	Inhibited lung tumor growth and prolonged survival in humanized mouse models	(37)
TCRdb2.0	Database of ~700 million TCR sequences for systematic analysis of γδ TCR diversity and tumor-reactive clonotypes	(211)
Dedifferentiated thyroid cancer γδTCR repertoire	Reduced diversity and increased clonal expansion in Vδ2neg and Vγ9neg subsets	(212)
Vδ2neg subset repertoire	Lower inter-individual overlap, suggesting private, tumor antigen-driven repertoire	(212)
Malignant γδ T cell clone monitoring	Enables MRD detection; clone dynamics correlate with chemotherapy response and relapse	(213)

**Figure 7:**
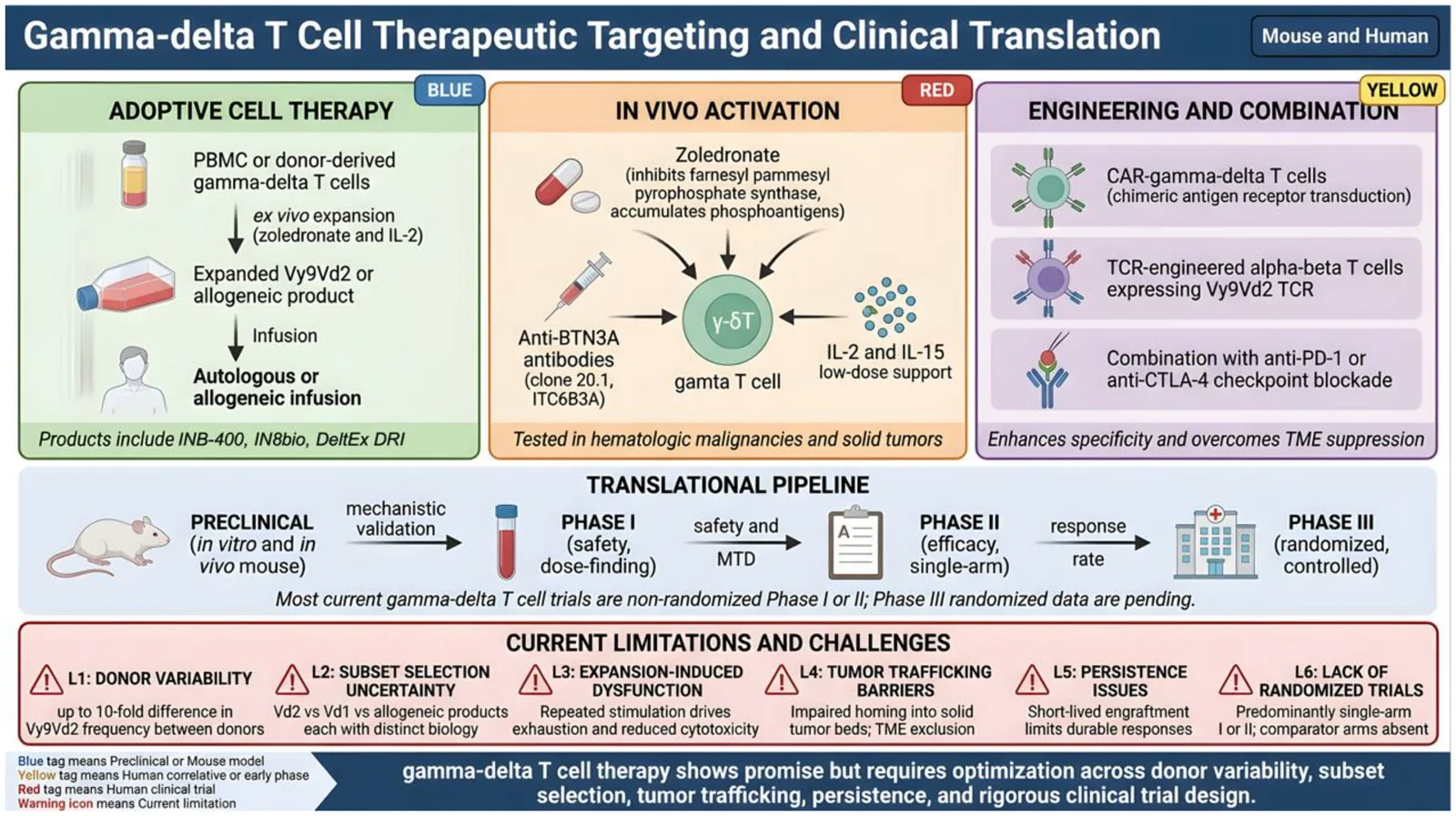
Therapeutic targeting and clinical translation of γδ T cells widescreen overview of γδ T cell -based cancer immunotherapy and its current limitations, organized into three horizontal bands. Band 1 (Strategies): three side-by-side panels cover (i) Adoptive Cell Therapy (ACT) -- donor PBMC isolation, *ex vivo* expansion with zoledronate/IL-2, and autologous or allogeneic infusion (representative products: GamaDelta, LymphoStrike, INB-400); (ii) *In vivo* activation -- zoledronate (farnesyl pyrophosphate synthase inhibition), agonistic anti-BTN3A antibodies (clones 20.1 and ITC6B3A), and low-dose IL-2/IL-15 support; and (iii) Engineering and Combination -- CAR-γδ T cells, αβ T cells transduced with Vγ9Vδ2 TCRs, and combinations with anti-PD-1/anti-CTLA-4 checkpoint blockade. Band 2 (Translational Pipeline): a four-stage flow from preclinical mouse models through Phase I, II, and III trials, with an explicit caveat that most current γδ T cell trials are non-randomized Phase I/II studies. Band 3 (Current Limitations and Challenges, red-bordered): six warning boxes addressing donor variability in Vγ9Vδ2 frequency, subset selection uncertainty, expansion-induced dysfunction, tumor-trafficking barriers, persistence issues, and the absence of randomized trials. Each strategy panel carries a color-coded evidence tag (blue preclinical/mouse; red human clinical trial; yellow human correlative/early phase) and a species banner.

### CAR-engineered γδ T cells

iPSC-derived CD19-targeted CAR T cells display γδ phenotype and inhibit lymphoma xenografts (214). CCR5-deficient γδ CD19 CAR T cells resist HIV-mediated depletion (215). For glioblastoma, a personalized cocktail CAR-Vδ1 T cell approach targets multiple heterogeneously expressed antigens using patient-derived GBOs with 88.46% cultivation rate (216). Engineered γδ T cells secreting tumor-targeting opsonins and IL-15Rα-IL-15 fusion proteins enhance efficacy in osteosarcoma (35). Chemical conjugation of anti-PD-L1 nanobodies via metabolic glycan labeling enhances tumor recognition and induces pyroptosis (217).

### γδ T cell-derived extracellular vesicle-based vaccines

γδ-T-EVs retain dual antitumor activities—direct cytotoxicity via FasL/TRAIL and immunostimulatory function via IFN-γ/CD40L (218). γδ-T-EVs loaded with tumor-associated antigens (TAAs) directly kill tumor cells and promote tumor-specific T-cell responses by delivering TAAs to APCs with adjuvant signals; allogeneic γδ-T-EVs show comparable efficacy to autologous, supporting off-the-shelf products (218).

### Combination therapies

In a single-patient case report, complete response to PD-1 blockade in Merkel cell carcinoma was associated with clonally expanded Vγ2Vδ1 T cells (219), although this observation requires validation in larger cohorts. Engineered anti-PD-1-secreting γδ T cells show enhanced efficacy (190). DNMTis upregulate NKG2D ligands and death receptors on tumor cells, potentiating Vδ1 cytotoxicity (220). Bispecific antibodies against CD20 × Vγ9 recruit Vγ9Vδ2 T cells to lymphoma (221). Siltuximab combined with γδ T cells counters IL-6-mediated immunosuppression in ovarian cancer (222).

### Clinical trial outcomes

In a non-randomized phase I trial, 132 late-stage cancer patients receiving allogeneic Vγ9Vδ2 T cell immunotherapy showed acceptable safety; an exploratory subgroup of 8 liver and 10 lung cancer patients receiving ≥5 infusions exhibited prolonged survival, although this *post-hoc* observation requires confirmation in randomized controlled trials (37). In LAPC, a small single-arm study of IRE plus allogeneic γδ T-cell infusion reported median OS of 14.5 vs 11 months and median PFS of 11 vs 8.5 months relative to historical controls (223); these results require validation in randomized trials. The combination reduced CTCs, elevated lymphocyte counts, upregulated NKG2D, and downregulated PD-1 (223).

### Challenges and future directions

The functional dichotomy necessitates subset selection; TCR-Vγδ usage distinguishes protumor from antitumor subsets (224). iPSC platform overcomes cell source limitations (214). The NF protocol enhances metabolic fitness and antitumor functions with clinical validation (37). Chemical engineering via metabolic glycan labeling offers rapid tumor targeting (217). γδ-T-EVs and hybrid exosome-photosensitizer platforms represent cell-free strategies (218, 225). IRE plus γδ T-cell infusion establishes clinical proof-of-concept for combining local ablative therapies with adoptive transfer (223). Activating STAT5B (N642H) mutations prevalent in γδ-PTCL can be targeted by JAK1/2 inhibitors (226). Comprehensive TCR databases enable biomarker discovery (211). γδTCR repertoire dynamics guide patient selection (212). MRD monitoring and immune reconstitution assessment are enabled by molecular techniques (213). Ongoing efforts to understand biology and rationally combine modalities will be essential (227).

## Limitations and challenges in γδ T cell therapeutics

Despite promising preclinical and early-phase clinical data, γδ T cell therapeutics face several major limitations. Donor variability: Vγ9Vδ2 frequencies vary up to 10-fold across donors, complicating standardization of allogeneic products. Subset selection: protumor γδT17 and antitumor γδIFN subsets must be rigorously distinguished, and TCR-Vγδ usage guides appropriate selection (224). Expansion-related dysfunction: prolonged ex vivo expansion induces exhaustion markers (PD-1, TOX1) and reduces cytotoxicity (182). Tumor trafficking: γδ T cells show variable homing to solid tumors; chemokine-receptor engineering (e.g., CCR2, CXCR2) may improve infiltration. Persistence: adoptively transferred γδ T cells typically persist less than two weeks; IL-15 support or iPSC-derived platforms may enhance durability (214). Lack of randomized trials: current clinical evidence is limited to small, non-randomized phase I/II studies, and definitive efficacy claims await randomized controlled trials. Emerging solutions include iPSC platforms to overcome donor variability (214), chemical engineering via metabolic glycan labeling for rapid tumor targeting (217), and γδ-T-EVs as off-the-shelf cell-free therapeutics (218). These limitations are consolidated visually in **Figure 7**.

## Conclusion and future perspectives

γδ T cells sit at the crossroads of innate and adaptive immunity, combining rapid innate-like effector function with antigen-specific TCR recognition. Their developmental programming, tissue-resident specialization, and dual protective/pathogenic roles in infection, cancer, and autoimmunity make them uniquely positioned immune sentinels.

Bridging innate and adaptive immunity: a unifying framework. The central thesis emerging from this review is that γδ T cells do not merely straddle the innate–adaptive boundary—they actively construct it. Their thymic effector programming equips them with rapid, TCR-triggered cytokine and cytotoxic responses that operate within hours (innate-like), yet their oligoclonal TCR repertoire, tissue-restricted ligand specificities, and adaptive-like memory in CMV and influenza demonstrate genuine adaptive features. Mechanistically, bridging occurs through three non-mutually exclusive routes: (i) direct antigen presentation to αβ T cells via inducible MHC class II and co-stimulatory molecules; (ii) cytokine-mediated instruction of αβ T, B, and myeloid cell differentiation (IL-17, IFN-γ, IL-21, IL-22); and (iii) innate-receptor integration of tissue stress signals (NKG2D, NKp30, CD16) that amplify downstream adaptive cascades. Exploiting these bridging routes—particularly the antigen-presenting capacity of Vγ9Vδ2 and Vδ1+ subsets—represents a tractable strategy for next-generation γδ T-cell therapeutics that combine rapid innate effector function with the specificity and durability of adaptive memory. The three bridging routes summarized in **Figure 1** encapsulate this framework.

Major Unanswered Questions. (1) The complete γδ TCR ligand repertoire remains undefined: while structural studies have resolved BTN3A1-mediated phosphoantigen sensing, BTNL3/8 interactions, and a small number of MHC-like complexes, the ligands recognized by most Vδ1+ and Vδ3+ clones are unknown. (2) The mechanisms that maintain long-term tissue residency without blood replenishment—particularly the transcriptional networks (Runx3, Bhlhe40, RORγt) and stromal cues sustaining residency—require systematic dissection (95). Human–mouse translational gaps remain substantial: many mechanisms defined in mice (e.g., Vγ5+ DETCs, Skint1 dependence) lack direct human correlates, and humanized models and cross-species single-cell atlases are needed to bridge this divide.

Priority Research Directions. (i) Structural immunology: cryo-EM of additional γδ TCR–ligand complexes, particularly for Vδ1/Vδ3 interactions with stress-induced self-proteins and antibody-like foreign antigen recognition (108). (ii) Therapeutic optimization: randomized clinical trials of allogeneic Vγ9Vδ2 therapy; CAR-γδ platforms targeting solid tumors; combination strategies with checkpoint blockade. (iii) Clinical biomarker development: TCR repertoire dynamics for patient stratification, MRD monitoring in γδ T-ALL, and polyspecific γδ TCR recognition (111) as a tool for immune monitoring. Resolving these questions will both advance fundamental immunology and unlock the therapeutic potential of γδ T cells.
